# C/EBPβ activation in vascular smooth muscle cells promotes hyperlipidemia-induced phenotypic transition and arterial stiffness

**DOI:** 10.1038/s41392-025-02196-w

**Published:** 2025-04-02

**Authors:** Jun Ma, Xiangyu Yang, Yanan Li, Xin Zhang, Kai Liu, Yong Peng, Si Wang, Rufeng Shi, Xingwei Huo, Xueting Liu, Xinran Li, Runyu Ye, Zhipeng Zhang, Changqiang Yang, Lu Liu, Dan Gao, Shanshan Jia, Lirong Sun, Xianghao Zuo, Qingtao Meng, Xiaoping Chen

**Affiliations:** Department of Cardiology, West China Hospital, Sichuan University, Sichuan, China

**Keywords:** Cardiovascular diseases, Cell biology

## Abstract

Arterial stiffness is a critical factor in cardiovascular and cerebrovascular events, yet clinical practice lacks specific therapeutic targets and biomarkers for its assessment. Hyperlipidemia closely correlates with arterial stiffness, and we observed elevated CCAAT/enhancer-binding protein β (C/EBPβ) expression in atherosclerotic mouse arterial walls. As the arterial medial layer predominantly consists of vascular smooth muscle cells (VSMCs), C/EBPβ‘s role in VSMCs under hyperlipidemia remains unclear. Our findings demonstrate that cholesterol-induced phenotypic transition of contractile VSMCs to macrophage-like cells coincides with C/EBPβ upregulation and activation. The activation of C/EBPβ is closely related to cellular assembly and organization, regulating the cytoskeleton via Disheveled-associated activator of morphogenesis 1 (Daam1). Conditional knockout of C/EBPβ in VSMCs of ApoE^−/−^ mice alleviated hyperlipidemia-induced vascular remodeling and reduced the elevation of aortic pulse wave velocity. Additionally, C/EBPβ-regulated cytokine platelet-derived growth factor-CC (PDGF-CC) is correlated with brachial-ankle pulse wave velocity in humans. These results indicate that the activation of C/EBPβ promotes the transition of VSMCs from a contractile phenotype to a macrophage-like phenotype by regulating morphological changes, and C/EBPβ activation contributes to hyperlipidemia-induced arterial stiffness. PDGF-CC exhibited a significant association with arterial stiffness and may serve as a promising indicator of arterial stiffness in humans. Our study reveals molecular mechanisms behind hyperlipidemia-induced arterial stiffness and provides potential therapeutic targets and biomarkers.

## Introduction

Arterial stiffness (or arteriosclerosis) refers to the reduced ability of arteries to accommodate blood flow, resulting from both functional and structural changes within the arterial system. These changes include diminished contractility of vascular smooth muscle cells (VSMCs), thickening of the vessel walls, and alterations in the composition of the extracellular matrix, etc.^1–3^. Arterial stiffness is closely associated with hypertension, stroke, coronary heart disease and other cardiovascular diseases (CVDs)^4–6^. The Framingham Heart Study recently reported that, for each standard deviation increase in carotid-femoral pulse wave velocity (PWV, a primary method for detecting arterial stiffness), the risk of CVDs increased by 1.2 times^6^. In recent years, there has been growing recognition that targeting arterial stiffness should be of high priority in order to reduce the risk of CVDs^2^. However, at present, there is a deficiency of identified therapeutic targets and biomarkers for effectively treating and preventing arterial stiffness.

Hyperlipidemia plays a pivotal role in inducing plaques and arterial stiffness^7–10^, and atherosclerosis is a specific form of arterial stiffness characterized by the formation of atherosclerotic plaques^11^. However, previous studies have predominantly focused on plaque formation, while the precise mechanisms underlying hyperlipidemia-induced arterial stiffness remain unclear. Studies have demonstrated that during the early stages of atherogenesis, VSMCs undergo a phenotypic switch from a contractile state to a macrophage-like state. Subsequently, the macrophage-like VSMCs exhibit lipid phagocytosis and convert into foam cells, thus promoting the formation of atherosclerotic plaques^12,13^. In in-vitro studies, the stimulation of VSMCs with 10 μg/ml methyl-β-cyclodextrin cholesterol (MBD-Chol) for 72 h induced contractile VSMCs into a macrophage-like phenotype^14,15^. In contractile VSMCs, there was high expression of proteins such as actin alpha 2 (ACTA2), Myosin Heavy Chain 11 (MYH11) and Calponin 1 (CNN1). Conversely, the macrophage-like phenotype exhibited high expression of CD68 and Lectin-galactose binding-soluble 3 (LGALS3)^16^. As VSMCs transition from a contractile to a macrophage-like phenotype, there is a marked decrease in their contractile capacity, accompanied by an increase in their phagocytic activity^16^. These observations suggest that, in the context of hyperlipidemia, the shift of contractile VSMCs to a macrophage-like phenotype may play a significant role in the development of arterial stiffness. However, the precise molecular mechanisms driving this transition in the presence of hyperlipidemia are still not fully understood.

CCAAT enhancer binding protein β (C/EBPβ) is a transcriptional regulator that influences the expression of various downstream genes. It is highly expressed in macrophages, and its knockout in bone marrow-derived macrophages has been shown to suppress the formation of hyperlipidemia-induced atherosclerotic plaques^17,18^. Previous studies have shown that C/EBPβ regulates VSMCs release of inflammatory factors and lipid uptake, functions typically associated with macrophages^19–21^. However, no research has addressed the potential role of C/EBPβ in VSMCs phenotype transition. On the other hand, in our ongoing investigation into the role of C/EBPβ in atherosclerosis, we observed notable expression of C/EBPβ not only in the plaques but also in the medial layer of the arteries. This layer is predominantly composed of VSMCs, and to date, the involvement of C/EBPβ in the arterial wall under high-fat conditions has not been explored. Therefore, we hypothesize that the expression of C/EBPβ may promote the transition of VSMCs from a contractile phenotype to a macrophage-like phenotype, and it may be associated with hyperlipidemia-induced arterial stiffness.

In this study, we observed high expression of C/EBPβ in the medial layer of the aortic arch and abdominal aorta of high-fat-induced atherosclerotic model mice. This high expression was closely associated with increased co-localization of CD68. In vitro, we used MBD-Chol to induce the transition of mouse primary aortic VSMCs from a contractile to a macrophage-like phenotype, and examined changes in C/EBPβ expression. Chromatin Immunoprecipitation sequencing (ChIP-seq) was used to assess functional changes in C/EBPβ and its involvement in the molecular mechanisms of VSMCs phenotype transition. We then constructed a hyperlipidemia-induced arterial stiffness model and conditionally knocked out C/EBPβ in VSMCs of ApoE^−/−^ mice. Using small animal ultrasound, we found that knocking out C/EBPβ in VSMCs effectively alleviated hyperlipidemia-induced arterial stiffness. Additionally, we screened the downstream secreted factor PDGF-CC regulated by C/EBPβ, and confirmed its correlation with arterial stiffness in human population study. Our study aims to explore the role of C/EBPβ in the expression of arterial VSMCs under a hyperlipidemia environment and to provide potential targets and biomarkers for the clinical diagnosis and treatment of hyperlipidemia-induced arterial stiffness.

## Results

### C/EBPβ expression increased in macrophage-like VSMCs, and knockdown of C/EBPβ inhibited phenotypic transition

**Fig. 1:**
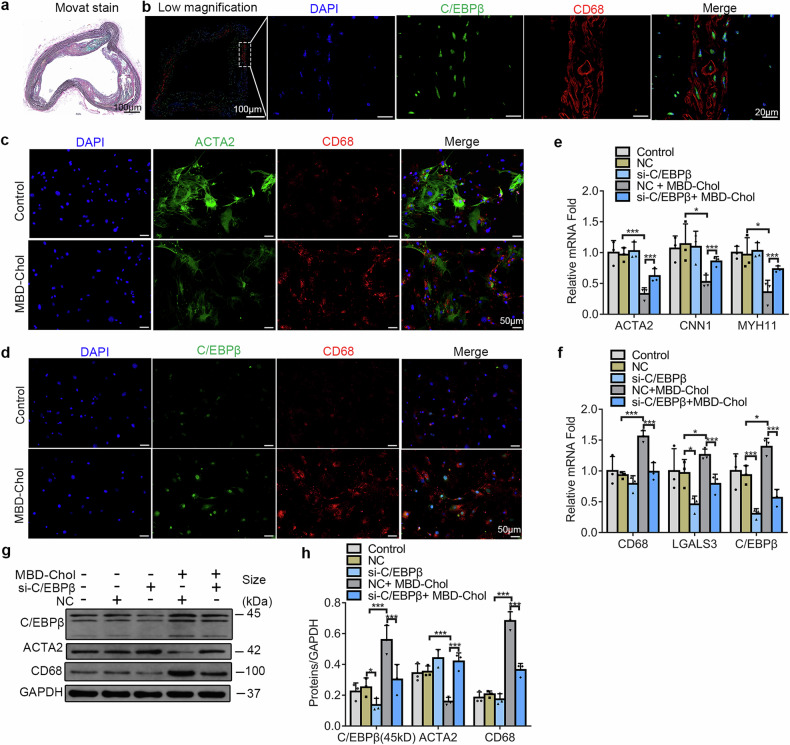
Knockdown of C/EBPβ expression inhibited MBD-Chol-induced transition of contractile VSMCs into a macrophage-like phenotype. **a** Modified Russell-Movat Pentachrome stain was used to show murine aortic arch tissue composition. Black for elastin fibers, yellow for collagen, blue/cyan for proteoglycan, red for collagen fibers, and black/purple for nuclei, scale bar = 100 μm. **b** Immunofluorescence staining was used to assess protein expression and localization in the murine aortas, blue (DAPI) for nuclei, green for C/EPBβ, and red for CD68 (representative images). The scale bars correspond to 100 μm for low-magnification images and 20 μm for high-magnification views. **c** Immunofluorescence staining was used to assess protein expression and localization in VSMCs, blue for nuclei, green for ACTA2, and red for CD68 (representative images), scale bar = 50 μm. **d** Immunofluorescence staining was used to assess protein localization and expression in VSMCs, blue for nuclei, green for C/EBPβ, and red for CD68 (representative images), scale bar = 50 μm. **e** mRNA levels of contractile phenotype-associated markers (ACTA2, CNN1, MYH11) in VSMCs, *N* = 3. **f** mRNA levels of macrophage-like phenotype-associated markers (CD68, LGALS3) and C/EBPβ in VSMCs, *N* = 3. **g** Protein expression levels detected by WB in VSMCs (representative images). **h** Statistical results of protein expression levels, *N* = 3. *p < 0.05 and ***p < 0.001. NC= scrambled siRNA group, si-C/EBPβ = C/EBPβ siRNA group. Segment of aortic arch was obtained for staining

To investigate the relationship between C/EBPβ and VSMCs phenotype transition, we first examined the expression of C/EBPβ in aortic sections from hyperlipidemic mice at different locations. In ApoE^−/−^ mice fed an atherosclerotic diet and with prominent plaque formation, we observed that cells with elevated C/EBPβ expression were present not only in the plaques but also in the medial layer of the aortic walls (Fig. 1a, b, Supplementary Fig. 1a–c). Next, we extracted primary aortic VSMCs from mice and induced phenotype transition in vitro using MBD-Chol, and knocked down C/EBPβ expression to observe changes in relevant markers. Following 72 h of stimulation with 10 μg/ml MBD-Chol, we noted a reduction in ACTA2 expression, along with an increase in C/EBPβ and CD68 expression, these changes in expression were often observed within the same cell (Fig. 1c, d). When C/EBPβ expression was knocked down, the suppression of ACTA2, CNN1, and MYH11 gene expression, as well as the increase in CD68 and LGALS3 gene expression triggered by MBD-Chol, was partially alleviated (Fig. 1e, f). Western blot results further confirmed that the knockdown of C/EBPβ partially mitigated the reduction in ACTA2 protein expression and the increase in CD68 protein levels induced by MBD-Chol stimulation (Fig. 1g, h). Our results indicate that C/EBPβ promotes the phenotype transition induced by MBD-Chol in VSMCs.

### Regulatory role of C/EBPβ in cytoskeleton in VSMCs following MBD-Chol stimulation

**Fig. 2:**
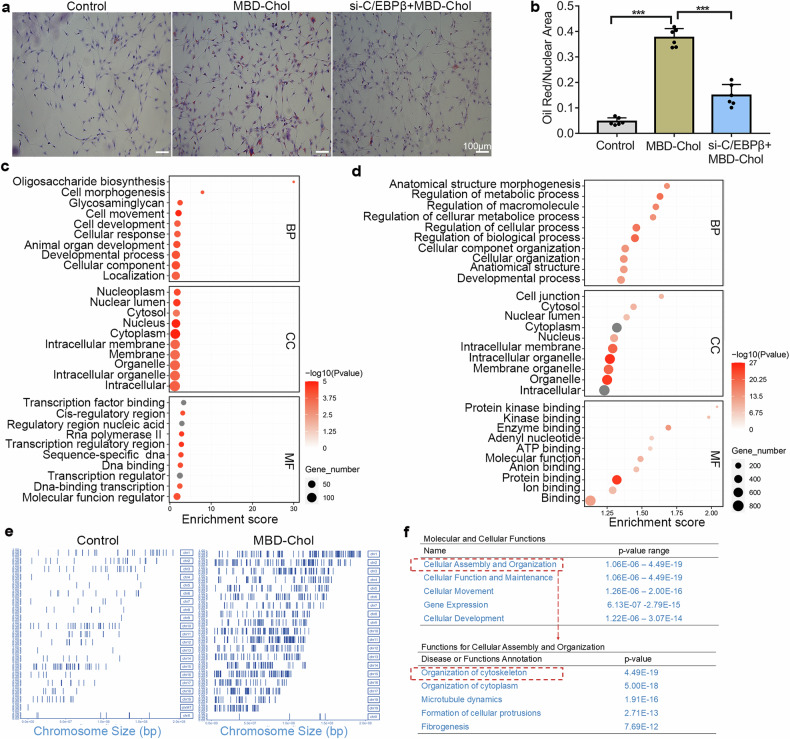
The role of C/EBPβ in the functional changes of VSMCs after MBD-Chol stimulation. **a** Oil Red O staining was used to detect lipid phagocytosis in VSMCs, the top image shows low magnification, the bottom image shows high magnification, scale bar=100μm, red for lipids. **b** Quantification of lipid area in Oil Red O staining, *N* = 6. **c** Functional enrichment analysis of ChIP-seq-identified C/EBPβ targets in control group (GO database). The change in red color from dark to light represents low to high P value, and the change in shape from small to large represents the increase in the number of related genes in the pathway from less to more, horizontal coordinates represent the enrichment score, the higher the score, the greater the effect, vertical coordinates represent the different functions. **d** Parallel analysis of MBD-Chol-treated specimens showing C/EBPβ‘s pathway engagement. **e** Distribution and location of peaks on different chromosomes, vertical coordinates represent the different chromosome numbers, horizontal coordinates represent the size of the chromosomes. **f** Classification of C/EBPβ-related functions in “Molecular and Cellular Functions” and “Cellular Assembly and Organization” by IPA, with *P*-values in ascending order from the top. *p < 0.05 and ***p < 0.001. NC = scrambled siRNA group, si-C/EBPβ = C/EBPβ siRNA group

In a high-fat environment, one of the key functions of macrophage-like phenotype VSMCs is phagocytosis of cholesterol^22^. To investigate the role of C/EBPβ in VSMCs, we assessed the lipid uptake function of VSMCs. After MBD-Chol treatment, their cholesterol phagocytic ability was enhanced. However, following C/EBPβ knockdown, this phagocytic ability decreased, indicating that changes in C/EBPβ expression affect the phagocytic ability of VSMCs (Fig. 2a, b).

C/EBPβ, as an enhancer-binding protein, plays a critical role in regulating gene transcription within cells. To explore the functional changes of C/EBPβ, we conducted ChIP-seq analysis followed by in-depth bioinformatics analysis. Compared to the control group, the number of genes bound by C/EBPβ was significantly elevated following stimulation with MBD-Chol (Fig. 2e). Comparative analysis using the GO database revealed differential functional enrichment, the control group showed baseline activity (Fig. 2c), MBD-Chol-treated specimens exhibited significant C/EBPβ involvement in anatomical structure morphogenesis (Fig. 2d). Using IPA, we annotated the downstream proteins regulated by C/EBPβ in VSMCs following MBD-Chol stimulation (Supplementary material Table S5). We categorized their functions into different groups based on the IPA database. Results showed that “Organization of Cytoskeleton” received the highest score, followed by “Organization of Cytoplasm”, “Microtubule Dynamics”, and “Formation of Cellular Protrusions” (Fig. 2f). The role of C/EBPβ in regulating anatomical structure morphogenesis may be key to the transition of VSMCs from a contractile phenotype to a macrophage-like phenotype.

### DAAM1 is a downstream protein of C/EBPβ and co-localizes with C/EBPβ

**Fig. 3:**
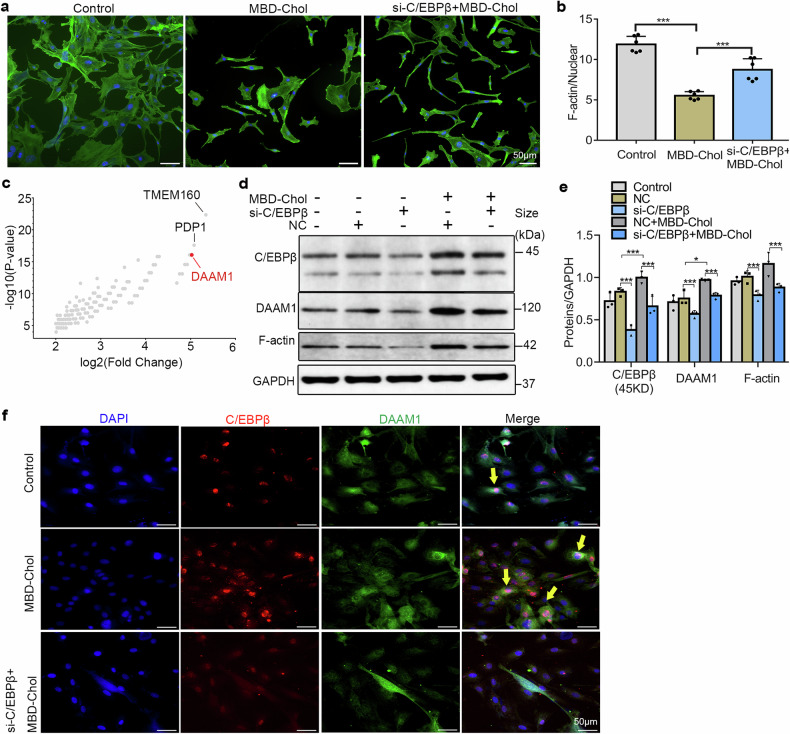
Regulatory role of C/EBPβ in cytoskeleton in VSMCs after MBD-Chol stimulation. **a** Phalloidin staining of VSMCs cytoskeleton in different groups. Blue for nuclei, and green for F-actin (representative images), scale bar = 50 μm. **b** Quantification of cytoskeleton area in Phalloidin staining, *N* = 6. **c** Results of ChIP-seq showing C/EBPβ-binding genes, horizontal coordinates represent fold change (log2), vertical coordinates represent P value (log 10). **d** Protein expression levels detected by WB in VSMCs (representative images). **e** Statistical results of protein expression levels, *N* = 3. **f** Immunofluorescence staining was used to assess protein localization and expression in VSMCs, blue for nuclei, green for Daam1, and red for C/EBPβ. Scale bar=50 μm. *p < 0.05 and ***p < 0.001. NC = scrambled siRNA group, si-C/EBPβ = C/EBPβ siRNA group

To confirm the role of C/EBPβ in cytoskeletal changes during VSMCs phenotypic transition, we performed phalloidin staining to observe alterations in the cytoskeleton. The results showed that after treatment with MBD-Chol, the quantity and morphology of F-actin filaments (the target protein of phalloidin, green) in VSMCs underwent significant changes. Following C/EBPβ knockdown, the amount of F-actin increased (Fig. 3a, b), suggesting that C/EBPβ is a crucial protein in regulating cytoskeletal changes during the transition of VSMCs to a macrophage-like phenotype. However, the specific molecular mechanism remains unclear. Subsequently, we identified Disheveled-associated activator of morphogenesis 1 (DAAM1) as a cytoskeleton-related protein with the highest fold change in expression in the VSMCs cytoplasm following MBD-Chol stimulation (Fig. 3c). DAAM1 is a cytoskeleton-related protein which localizes with F-actin. The silencing of DAAM1 can lead to severe defects in filopodial number, integrity, and architecture^23,24^. According to our results, the expression of DAAM1 & F-actin was up-regulated following MBD-Chol stimulation, while the knockdown of C/EBPβ expression partially suppressed the increase in DAAM1 expression (Fig. 3d, e). Moreover, our fluorescence results revealed clear co-localization of DAAM1 with C/EBPβ (Fig. 3f). Combined with the ChIP-seq findings, it suggests that C/EBPβ directly binds to the DAAM1 gene, regulating its expression and subsequently influencing cytoskeletal alterations.

### Feeding ApoE^−/−^ with a 60% high-fat diet for 14 weeks induced arterial stiffness without plaque formation

Based on the results above, we believe C/EBPβ may play an important role in contributing to arterial stiffness and reduced vascular contractility, we need to validate the role of C/EBPβ in hyperlipidemia-induced arterial stiffness. Considering the potential involvement of complex molecular mechanisms in arterial stiffness during plaque formation (especially the effect of C/EBPβ activation in bone marrow-derived macrophages^17,25^), we deemed it necessary to investigate the role of C/EBPβ in hyperlipidemia-induced arterial stiffness using a model without plaque formation. When constructing atherosclerosis models using ApoE^−/−^ mice, additional cholesterol is typically required. In our previous work, we fed ApoE^−/−^ mice with a 60% high-fat diet without cholesterol, and found it led to concentric vascular remodeling (medial thickening) and significantly prolonged the time for plaque formation. We believe that additional cholesterol is necessary in a high-fat diet to accelerate plaque formation. To specifically study arterial stiffness without the confounding effects of plaque development, we fed ApoE^−/−^ mice with a 60% high-fat diet without additional cholesterol (HF group) to establish a model of arterial stiffness without concurrent plaque development. Pulse wave velocity (PWV) measured by ultrasound is the gold standard for clinical assessment of arterial stiffness^2^.Using small animal ultrasound, we conducted measurement of Aortic pulse wave velocity (aPWV) and the vasoactive diameter ratio (minimum diameter during vasoconstriction [Dmin] / maximum diameter during vasodilation [Dmax]) in mice to assess arterial stiffness and aortic contractile function.

**Fig. 4:**
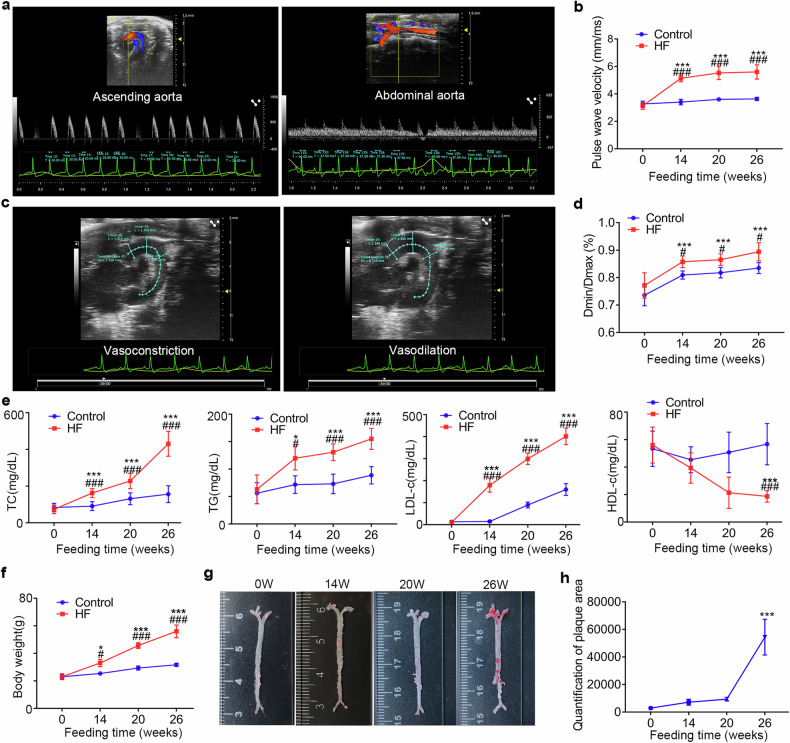
Changes in lipids levels, arterial stiffness and plaque formation at different times in ApoE^−/−^ mice fed with 60% high-fat diet. **a** Small animal ultrasound was used to assess aPWV in vivo (representative images). **b** Statistical results of changes in aPWV of mice at different feeding times, *N* = 10. **c** Small animal ultrasound was used to assess aortic contractile function in vivo (representative images). **d** Statistical results of changes in aortic contractile function of mice at different feeding times, *N* = 10. **e** Changes in blood lipids levels of mice at different feeding times, *N* = 10. **f** Changes in body weight of mice at different feeding times, *N* = 10. **g** Plaque formation in murine aortas at different feeding times (representative images). **h** Statistical results of plaque formation in murine aortas at different feeding times, *N* = 10. TC total cholesterol, TG triglycerides, LDL-c low-density lipoprotein cholesterol, HDL-c high-density lipoprotein cholesterol. Control=ApoE^−/−^ mice fed with chow food, HF (ApoE^−/−^) ApoE^−/−^ mice fed with a 60% high-fat diet. *p < 0.05 and ***p < 0.001 compared with 0-week, ^#^*p* < 0.05 and ^###^*p* < 0.001 compared with the control group

Compared with the control group (ApoE^−/−^ mice on a chow diet), aPWV and vasoactive diameter ratio in the HF group significantly increased at 14 weeks, and maintained the trend of increasing at 20 weeks and 26 weeks (Fig. 4a–d). However, no significant difference was observed in aPWV or vasoactive diameter ratio between the HF and HFHC groups at 14 weeks (Supplementary Fig. 2a, b). Total cholesterol (TG), triglyceride (TC), low-density lipoprotein cholesterol (LDL-c) and body weight (BW) of mice in the HF group were significantly higher than those in the control group at 14 weeks (Fig. 4e, f). High-density lipoprotein cholesterol (HDL-c) of mice in the HF group was significantly lower than those in the control group at 26 weeks, but not at 14 weeks or 20 weeks (Fig. 4e). When compared the HF group with the HFHC group at 14 weeks, TC and LDL-c levels were significantly higher in the HFHC group (Supplementary Fig. 2d), while there were no differences in BW or TG between the two groups (Supplementary Fig. 2c, d). Notably, significant plaques were observed in the HF group at 26 weeks, but not at 14 weeks or 20 weeks (Fig. 4g, h). In contrast, the HFHC group developed significant plaques at 14 weeks (Supplementary Fig. 2e, f). These results suggest that a high-fat diet without additional cholesterol (HF group) induces significant hyperlipidemia and arterial stiffness in ApoE^−/−^ mice at 14 weeks.

### C/EBPβ knockout in VSMCs of ApoE^−/−^ mice reduced the arterial stiffness induced by hyperlipidemia

**Fig. 5:**
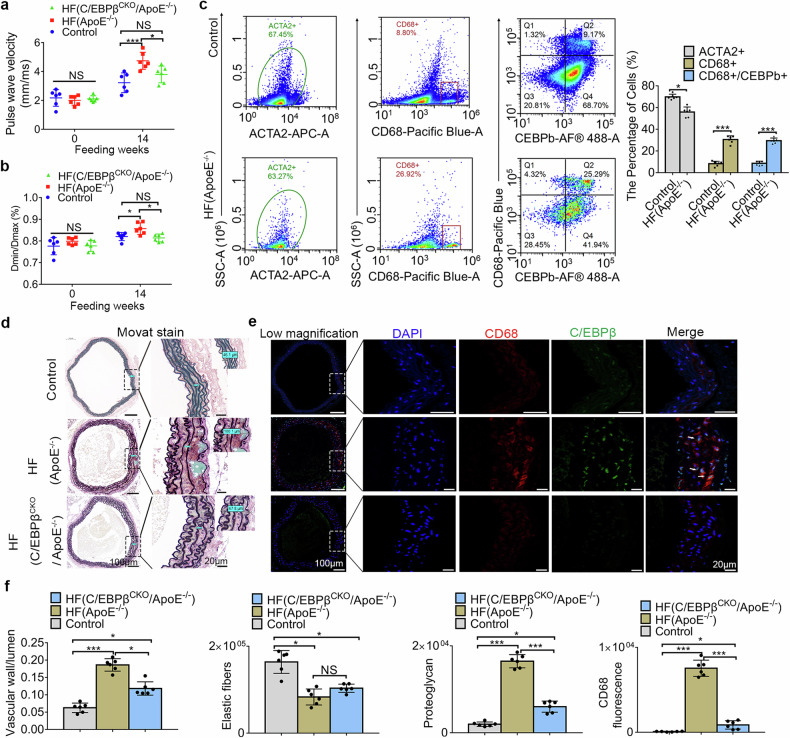
Knockout of C/EBPβ in VSMCs alleviated hyperlipidemia-induced arterial stiffness. **a** Statistical result of changes in aPWV, *N* = 6. **b** aortic contractile function in vivo, *N* = 6.**c** Flow cytometry results of the Control group and the HF (ApoE^−/−^) group, *N* = 6. **d** Modified Russell–Movat Pentachrome stain was used to show murine arterial tissue composition. Black for elastin fibers, yellow for collagen, blue/cyan for proteoglycan, red for collagen fibers, and black/purple for nuclei (representative images), scale bars correspond to 100 μm for low-magnification images and 20 μm for high-magnification views. **e** Immunofluorescence staining was used to assess protein expression and localization in the murine aortas, blue for nuclei, green for C/EBPβ, and red for CD68 (representative images), scale bars correspond to 100 μm for low-magnification images and 20 μm for high-magnification views. **f** From left to right, respectively, are statistical results of vascular wall/lumen measured ex vivo, *N* = 6, statistical results of elastic fibers, *N* = 6; Statistical results of proteoglycan, *N* = 6, Statistical results of CD68 fluorescence area, *N* = 6. ACTA2 + = ACTA2-positive cells, CD68 + = CD68-positive cells, CD68 + /CEBPb + = CD68/C/EBPβ double-positive cells. Control = ApoE^−/−^ mice fed with chow food, HF (ApoE^−/−^) = ApoE^−/−^ mice fed with a 60% high-fat diet, HF (C/EBPβ^CKO^/ApoE^−/−^) = ApoE^−/−^/C/EBPβ^fl/fl-^-Tagln^cre^ mice fed with a 60% high-fat diet. *p < 0.05 and ***p < 0.001. Segment of aortic arch was obtained for staining

To investigate the role of C/EBPβ in arterial VSMCs in hyperlipidemia-induced arterial stiffness, we constructed ApoE^−/−^ mice with VSMCs conditional knockout of C/EBPβ (C/EBPβ^CKO^/ApoE^−/−^). Using small animal ultrasound, pathology, flow cytometry, and other techniques, we compared the arterial stiffness of aortic VSMCs in hyperlipidemic mice with and without C/EBPβ knockout. After 14 weeks of feeding, a significant increase in aPWV was observed in the HF group compared to the control group, conditional knockout of C/EBPβ in VSMCs alleviated the hyperlipidemia-induced rise in aPWV (Fig. 5a). Similarly, the vasoactive diameter ratio in the HF group was significantly higher than that in the control group, and the conditional knockout of C/EBPβ in VSMCs mitigated these changes (Fig. 5b). Flow cytometry analysis revealed that, after 14 weeks of high-fat feeding, the number of ACTA2-positive cells in the aortas of ApoE^−/−^ mice was lower compared to those on a normal diet, while CD68-positive cells and CD68/C/EBPβ double-positive cells were significantly increased (Fig. 5c). However, it is regrettable that in conditionally C/EBPβ-knockout ApoE^−/−^ mice under high-fat feeding, very few viable cells were obtained from digested aortic tissue, making it impossible to detect changes in the relevant positive cell populations. Similarly, when we attempted single-cell sequencing, this issue persisted, and it was not possible to obtain sufficient viable aortic cells from the HF (C/EBPβ^CKO^/ApoE^−/−^) group for detection (Supplementary Fig. 3d).

In pathological and tissue immunofluorescence staining, the HF group showed a notable increase in the vessel wall/lumen ratio and proteoglycan content (green and blue) compared to the control group (Fig. 5d, f). Conditional knockout of C/EBPβ in VSMCs of ApoE^−/−^ mice (C/EBPβ^CKO^/ApoE^−/−^ group) reduced the thickening of the vessel wall and the elevation of proteoglycan content induced by hyperlipidemia (Fig. 5e, f). Elastic fibers were significantly reduced in both the HF and HF(C/EBPβ^CKO^/ApoE^−/−^) groups when compared to the control group. Interestingly, no significant difference in elastic fiber changes was observed between the HF and HF(C/EBPβ^CKO^/ApoE^−/−^) groups (Fig. 5e, f). Additionally, fluorescence staining showed a marked increase in fluorescence intensity for CD68 in the HF group compared to the control group (Fig. 5e, f). Colocalization of CD68 and C/EBPβ fluorescence was observed in the arterial media, and their localization was close to the site of proteoglycan deposition (orange arrows, Fig. 5d, e). In contrast, the HF(C/EBPβ^CKO^/ApoE^−/−^) group showed significantly lower fluorescence intensity of CD68 compared with the HF group (Fig. 5e, f). Our results suggest that C/EBPβ is a key factor in hyperlipidemia-induced arterial stiffness, and this effect may be closely related to VSMCs phenotype transition.

### PDGF-CC was found to be increased in VSMCs after MBD-Chol treatment

To address the lack of effective biomarkers for arterial stiffness in clinical practice, we screened the downstream secretory factor genes that C/EBPβ can bind to in ChIP-seq after its high expression, and performed upstream and downstream validation. Additionally, we used RNA sequencing data from human coronary artery VSMCs provided by another research team to further verify whether these results are applicable to human populations.

**Fig. 6:**
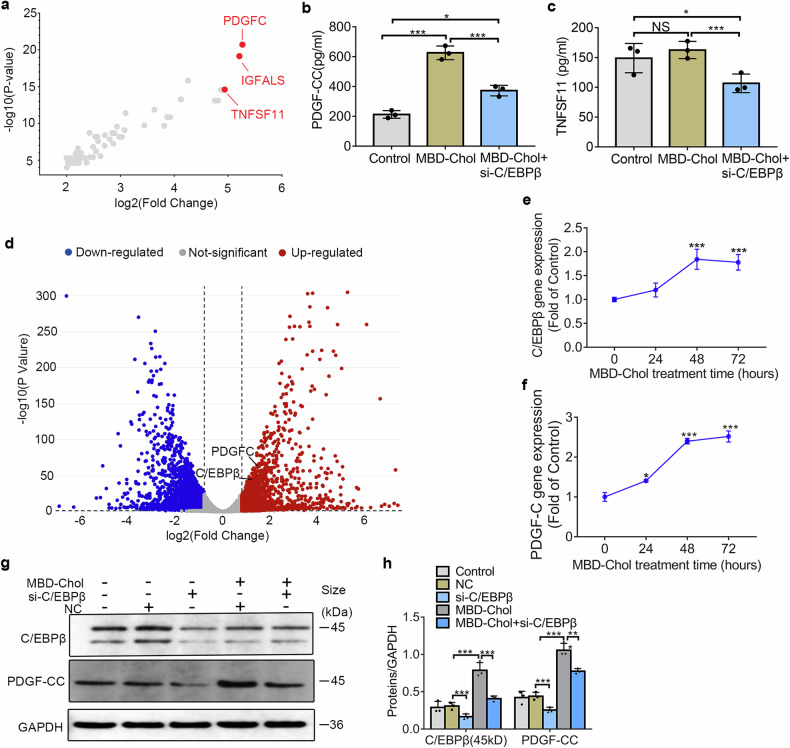
Downstream secretory factor regulated by C/EBPβ—PDGF-CC levels increased. **a** Screening for exocytosis factor genes that could be bound by C/EBPβ in VSMCs after MBD-Chol stimulation, horizontal coordinates represent fold change (log_2_), vertical coordinates represent P value (log _10_). **b** Levels of PDGF-CC in murine VSMCs supernatants, *N* = 3. **c** Levels of TNFSF11 in murine VSMCs supernatants, *N* = 3. **d** Volcano plot showed the difference in genes expression levels in human VSMCs after 10 μg/ml MBD-Chol treatment for 0 h and 72 h, blue for genes down-regulated expression, red for genes up-regulated expression, horizontal coordinates represent fold change (log_2_), vertical coordinates represent *P* value (log_10_), the threshold for *P*-value is 0.05, the threshold for fold change is 0.8. **e** C/EBPβ gene expression levels in VSMCs of human species. **f** PDGF-C gene expression levels in VSMCs of human species. **g** Protein expression levels detected by WB in murine VSMCs (representative images). **h** Statistical results of protein expression levels, *N* = 3. *p < 0.05 and ***p < 0.001

We identified *PDGF-C, IGFALS*, and *TNFSF11* as the top three genes with the highest fold change among those that could be secreted into the extracellular environment (Fig. 6a). Using ELISA, we measured the levels of secreted factors in the supernatant of VSMCs. The results indicated that MBD-Chol treatment increased the levels of PDGF-CC, while knockdown of C/EBPβ alleviated this effect (Fig. 6b). This phenomenon was also confirmed at the protein level (Fig. 6g, h). However, treatment with PDGF-CC did not lead to an increase in C/EBPβ expression or induce phenotype transition in VSMCs (Supplementary Fig 4c). Besides, MBD-Chol did not elevate the levels of TNFSF11(Fig. 6c) and IGFALS was not tested due to the unavailability of ELISA kits. Analysis of RNA sequencing data obtained from a previous study (https://ncbi.nlm.nih.gov/geo/query/acc.cgi?acc=GSE163244)^26^ revealed significant up-regulation of C/EBPβ and PDGF-C gene expression in human VSMCs after MBD-Chol treatment (Fig. 6d). Both C/EBPβ and PDGF-C gene expression showed an increasing trend, with significant changes observed at 48 h for C/EBPβ (Fig. 6e), and at 24 h for PDGFC (Fig. 6f). Therefore, our results suggest that PDGF-CC, a downstream secretory factor regulated by C/EBPβ, may serve as a potential biomarker for high-fat-induced arterial stiffness.

### Serum PDGF-CC levels were correlated with brachial-ankle pulse wave velocity and served as a potential marker for distinguishing individuals with arterial stiffness

**Fig. 7:**
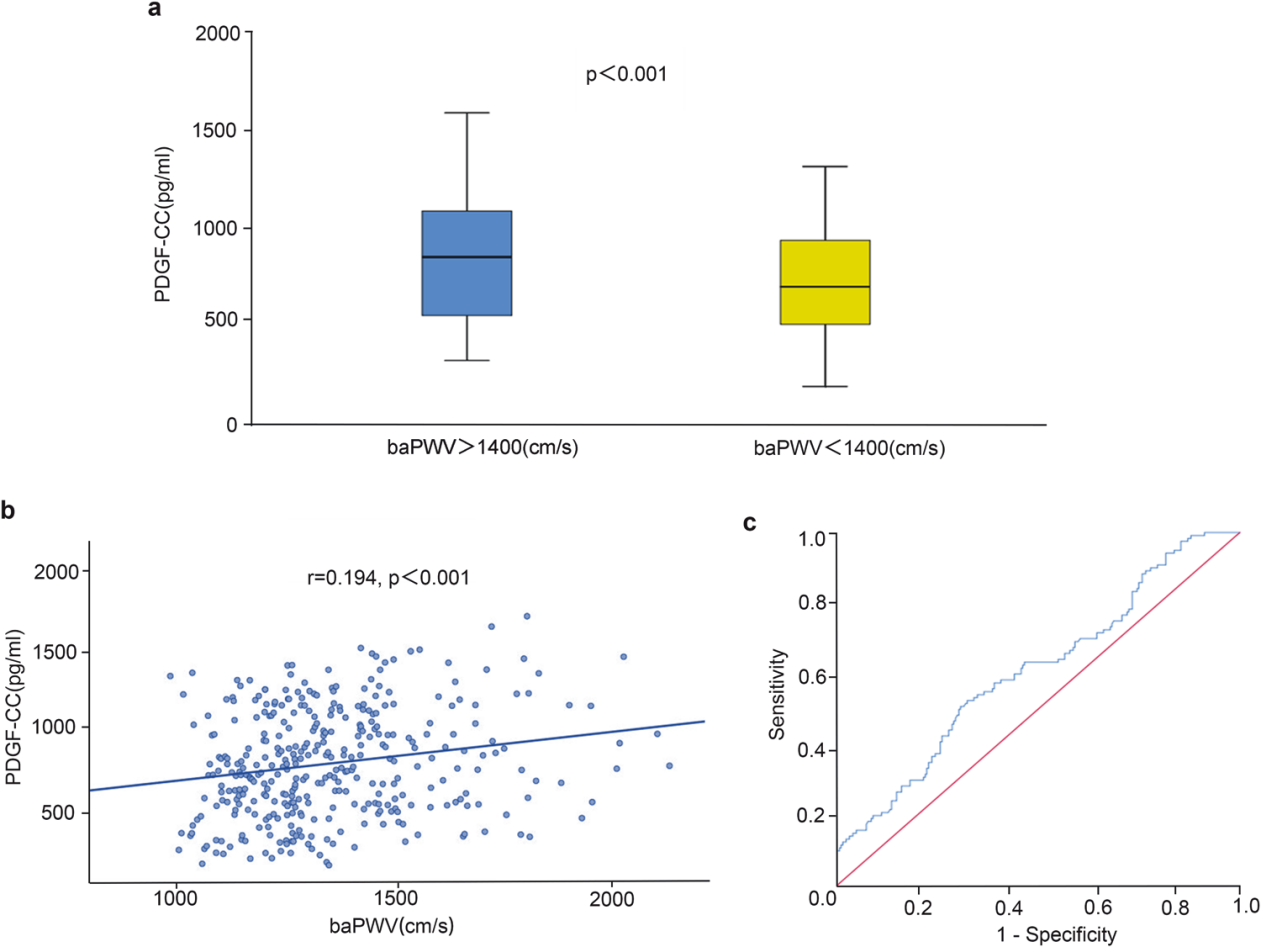
Correlation between PDGF-CC and baPWV in population. **a** Comparison of plasma PDGF-CC levels between participants with baPWV ≥1400 cm/s and baPWV < 1400 cm/s. **b** Relationship between plasma PDGF-CC levels and baPWV in the whole cohort. **c** Receiver operative characteristic curves for distinguishing baPWV ≥1400 cm/s by plasma PDGF-CC levels

To evaluate the reliability of PDGF-CC as a biomarker for arterial stiffness, we conducted a cross-sectional correlation study in a human population. The clinical baseline characteristics of participants with brachial-ankle pulse wave velocity (baPWV) ≥ 1400 cm/s and baPWV < 1400 cm/s are summarized in Table 1. The baPWV ≥1400 cm/s group had significantly higher values for SBP, DBP, FBG, TC, LDL-C, and UA compared to the baPWV < 1400 cm/s group. Additionally, the plasma PDGF-CC levels were notably higher in the baPWV ≥1400 cm/s group (median 847.73 pg/ml vs. 679.49 pg/ml, *P* < 0.001, Fig. 7a). Spearman correlation analysis (Fig. 7b) revealed a positive relationship between plasma PDGF-CC concentration and baPWV (*r* = 0.194, *P* < 0.001) across all subjects. After adjusting for potential confounders, the plasma PDGF-CC level remained positively associated with baPWV in the entire cohort, with partial correlation coefficients of 0.161 in model 2 and 0.148 in model 3 (Supplementary Table 2). Besides, the ROC curves demonstrated the ability of the plasma PDGF-CC level to distinguish arterial stiffness (Fig. 7c). The AUC for arterial stiffness based on the plasma PDGF-CC level was 0.608 (95%CI: 0.546–0.670). The YI, PPV, and NPV of the plasma PDGF-CC level cut-off value of 826.02 pg/ml for baPWV ≥1400 cm/s were 20.0, 44.6, and 74.0%, respectively (Supplementary Table 3). The above results indicate that PDGF-CC is significantly correlated with arterial stiffness in the human population, and it may serve as a reliable indicator for predicting changes in arterial stiffness.

**Table 1:** Clinical baseline characteristics of the participants in the population study

Variables	BaPWV ≥1400 cm/s *N* = 120	BaPWV <1400 cm/s *N* = 240	*P*
Age (years old)	44.50 (33.50, 51.00)	46.00 (35.00, 51.00)	0.603
Female (%)	37.50	37.50	1
History of Hypertension (%)	3.30	0.40	0.044
History of diabetes (%)	0	0.80	0.554
Current smoking (%)	25.80	31.30	0.288
Alcohol drinking (%)	38.30	34.60	0.484
BMI (kg/m^2^)	24.50 (22.37, 26.95)	23.57 (21.32, 25.87)	0.081
SBP (mmHg)	129.50 (117.75, 147.17)	117.50 (110.00, 126.92)	<0.001
DBP (mmHg)	86.33 (76.42, 93.33)	75.67 (71.00, 82.00)	<0.001
FBG (mmol/L)	5.25 (4.92, 5.74)	5.14 (4.87, 5.46)	0.065
TG (mmol/L)	1.15 (0.76, 2.09)	1.08 (0.78, 1.66)	0.171
TC (mmol/L)	5.00 (4.30, 5.58)	4.80 (4.30, 5.30)	0.032
HDL-C (mmol/L)	1.40 (1.17, 1.57)	1.43 (1.23, 1.59)	0.434
LDL-C (mmol/L)	3.14 ± 0.72	2.87 ± 0.64	<0.001
CR (μmol/L)	65.50 (53.25, 73.75)	65.00 (54.00, 75.00)	0.605
UA (μmol/L)	339.00 (273.25, 431.50)	322.00 (268.25, 380.00)	0.04
PDGF-CC (pg/ml)	847.73 (512.93, 1108.44)	679.49 (463.90, 944.05)	<0.001
BaPWV (cm/s)	1516.50 (1445.25, 1663.75)	1233.50 (1143.00, 1294.00)	<0.001

Values are expressed as mean ± SD, median (interquartile range), or number of participants (%)

*BMI* body mass index, *SBP* systolic blood pressure, *DBP* diastolic blood pressure, *FBG* fasting blood glucose, *TG* triglyceride, *TC* total cholesterol, *HDL-C* high-density lipoprotein cholesterol, *LDL-C* low-density lipoprotein cholesterol, *CR* Creatinine, *UA* uric acid, *PDGF-CC* Platelet-derived growth factor CC, *BaPWV* brachial-ankle pulse wave velocity

## Discussion

Our study demonstrated that when stimulated with 10 μg/ml MBD-Chol for 72 h, VSMCs underwent a transition from a contractile phenotype into a macrophage-like phenotype (Fig. 1c–h), and it is consistent with previous findings^14,27^. We observed an increase in C/EBPβ expression, which co-localized with CD68 in VSMCs, knockdown of C/EBPβ in VSMCs partially reversed the MBD-Chol-induced reduction in contractile-related markers and the upregulation of macrophage-like phenotype-related markers (Fig. 1e–h). These findings indicate that C/EBPβ plays a critical role as a regulatory protein in the transition of contractile VSMCs to a macrophage-like phenotype. Another transcription factor, Krüppel-Like Factor 4 (KLF4), has been shown to play a critical role in the phenotypic transition of VSMCs^28^, it is speculated that C/EBPβ might influence the phenotypic transition of VSMCs through the regulation of KLF4^16^. Interestingly, we found in ChIP-seq results as well as at the gene and protein levels that C/EBPβ can directly regulate the expression of Lgals3 (Fig. 1f, Supplementary Table S5). However, while Lgals3 (also known as galectin-3) has been considered as a macrophage-like phenotype marker, it has also been associated with osteoblast-like phenotypes^29^. Whether C/EBPβ is related to osteoblast-like phenotypic smooth muscle cells requires further investigation.

Our study showed that C/EBPβ function was activated in macrophage-like VSMCs (Fig. 2). The primary molecular and cellular functions performed by C/EBPβ were closely associated with Cellular Assembly and Organization (Fig. 2c–e). We also found that MBD-Chol resulted in enlarged nuclei, shortened skeletons and disorganized arrangements in VSMCs (Fig. 2a). Besides, we observed horseshoe-shaped megakaryocytes in the images (Fig. 2a), which might be a result of VSMCs phenotypic transition, but we cannot exclude the possibility of contamination from a very small number of mononuclear cells, as our cells were extracted and processed from the mouse aorta. However, overall, we found that after MBD-Chol treatment, the ability of VSMCs to uptake cholesterol was significantly increased, and this phenomenon could be inhibited by C/EBPβ knockdown. Our study suggests that C/EBPβ may be a key regulatory molecule in the early stages of atherosclerosis, where VSMCs phagocytize lipids and transition into foam cells. A recent study reported that MBD-Chol induced increased cytoskeleton orientation and stiffness in VSMCs^30^, but the study did not discuss the phenotypic transition of VSMCs and used a different approach with MBD-Chol treatment for only 30 min^30^. Furthermore, our findings suggest that C/EBPβ plays a significant role in mediating the MBD-Chol-induced cytoskeletal changes, which has not been reported before. In the subsequent analysis of ChIP-seq data, we identified DAAM1 as one of the proteins with the most significant fold change in the VSMCs cytoplasm following MBD-Chol stimulation (Fig. 3c). DAAM1 is closely associated with cytoskeleton organization by affecting the F-actin assembly and elongation^23,31,32^, our WB results indicate that DAAM1 is a downstream molecule regulated by C/EBPβ (Fig. 3d, e). C/EBPβ may exert its function on cytoskeleton alterations and VSMCs phenotypic transition by regulating the expression of DAAM1 (Fig. 3).

Current studies investigating hyperlipidemia-induced arterial stiffness often employ models with concurrent atherosclerotic plaque formation^33^. Nevertheless, the exact relationship between plaque formation and arterial stiffness remained to be elucidated. The activation of C/EBPβ in bone marrow-derived macrophages during atherogensis (e.g., inflammatory factor release^17,25^) might overlap the effect of C/EBPβ activation in macrophage-like VSMCs. Therefore, we believe that studying the role of C/EBPβ in hyperlipidemia-induced arterial stiffness would be better accomplished in a model without plaque formation.

We observed that feeding ApoE^−/−^ mice with a 60% fat diet (HF group) instead of an atherosclerotic diet (HFHC group) delayed the appearance of plaques (Fig. 4g, h). The varying changes in blood lipid levels induced by different diets may be a key factor affecting the inconsistency in the timing of pathological changes, a recent study also discussed the impact of high-fat dietary habits on plaque development^34^. Our findings suggest that levels of TG, but not TC or LDL-c (Fig. 4e, Supplementary Fig. 2d), might have an effect on arterial stiffness, which was similar to the results of a previous population study^9^. Additionally, a high-fat diet primarily consists of triglycerides. We treated VSMCs with 100 μM oleic acid and palmitic acid^35^ for 72 h. Results showed that compared to the control group, there were no significant changes in ACTA2 and CD68 expression in the oleic acid and palmitic acid groups (Supplementary Fig. 4d). Our results demonstrated that conditional knockout of C/EBPβ in VSMCs alleviated hyperlipidemia-induced arterial stiffness, as evidenced by an ameliorative increase in aPWV, an improvement in arterial diastolic function, reduction in vessel wall thickness, attenuated decrease in elastic fibers and alleviated increase in proteoglycans (Fig. 5d, f). The effect of C/EBPβ on arterial stiffness may be closely related to its role in regulating the phenotypic transition of VSMCs, since contractile VSMCs contained a large amount of ATCA2 and had a strong contractility. Previous studies have shown that estrogen plays a protective role in high-fat-induced vascular diseases^34,36^. Similarly, in our study, we found that the rate of arterial stiffness increase in female mice is slower than in male mice (Supplementary Fig. 3a, b). Still, C/EBPβ knockout reduces the increase of arterial stiffness induced by a high-fat diet in female mice.

Regarding the exploration of the role of C/EBPβ in VSMCs phenotypic transition in mice subjected to a high-fat diet, we planned to conduct flow cytometry or single-cell sequencing on mouse aortas to validate our findings. In our flow cytometry results, the HF group exhibited a decrease in ACTA2-positive cells in the aorta compared to the control group, while CD68-positive cells and CD68/C/EBPβ double-positive cells showed a significant increase (Fig. 5c). Nevertheless, we found that in C/EBPβ^CKO^/APOE^−/−^ mice, it seems challenging to obtain enough cells from the mouse aorta using the conventional digestion method (collagenase IV or collagenase IV + collagenase I), while Sufficient cells can be obtained when digesting mice with either Control or HF group (Supplementary Fig. 3d). According to previous literature, C/EBPβ is closely related to cellular glucose and lipid metabolism^37^, and about one-third to half of the C/EBPβ knockout mice die within 24 h after birth^38^. Therefore, after hyperlipidemia stimulation, VSMCs with C/EBPβ knocked out may be more fragile than normal VSMCs, making it difficult for us to isolate viable cells. Although knocking out C/EBPβ in VSMCs in this study alleviated arterial stiffness, we believe this gene, as a critical transcription factor located in the nucleus, may not be suitable as a direct therapeutic target for treating diseases.

The results of ChIP-seq analysis showed that PDGF-CC were important downstream secretory factors regulated by C/EBPβ, and it could potentially serve as markers for arterial stiffness (Fig. 6). Based on the RNA-seq data from a previous study^26^, we found a significant increase in C/EBPβ and PDGF-C gene expression after 72 h of MBD-Chol treatment in human VSMCs (Fig. 6d–f). We hypothesized that PDGF-CC might be associated with arterial stiffness in population. Previous studies have shown that PDGF-BB can promote the phenotypic transition of VSMCs^39^. The role of PDGF-CC in arterial stiffness remains unknow, using the concentrations and treatment durations referenced in earlier studies^40^, PDGF-CC did not induce the transition of VSMCs phenotype (Supplementary Fig. 4c). Through literature review, we found that the receptors for PDGF-CC, PDGFRα/β, are highly expressed on VSMCs^41^. PDGF-CC binding to PDGFRα/β can activate the NF-κB/interleukin-6 (IL-6) pathway^42^. IL-6 plays a key role in promoting the formation of atherosclerotic plaques^43^, we hypothesize that the increased secretion of PDGF-CC by macrophage-like VSMCs may triggers the NF-κB/IL-6 pathway in contractile VSMCs, leading to the progression from arterial wall stiffness to plaque formation.

In our population study, we found elevated serum PDGF-CC levels in participants with baPWV > 1400 cm/s. Furthermore, PDGF-CC levels were positively correlated with baPWV and could potentially serve as a marker for identifying individuals with arterial stiffness (Fig. 7). To our knowledge, this finding has not been reported in previous research. However, both the correlation and the distinguishing ability were not very strong, which could be attributed to the population selection. In the present study, a natural population cohort was used for baPWV testing. In addition, patients with high cardiovascular risks or history of cardiovascular events were also excluded before the matching process. Nevertheless, C/EBPβ was predominantly activated under hyperlipidemia conditions in our basic research part. This might account for the relatively weak correlation coefficient.

This study has some limitations. This study utilized the Tagln (SM22 alpha)-Cre system to knockdown C/EBPβ in VSMCs. Tagln is also expressed in myeloid cells and adipocytes, which may affect result interpretation due to potential off-target effects. Subcultured VSMCs rapidly dedifferentiate and lose their contractile phenotype, resulting in significantly reduced expression of contractile proteins, a lineage tracing-based cell sorting method would provide a more reliable approach for these experiments. Finally, the method we used to assess arterial stiffness is based on the principle of cfPWV, which is consistent with clinical practice. This method has been reported in previous study^44^, we improved the procedure by measuring the complete vessel ex vivo after collection (Supplementary Fig. 7). However, there is currently no standardized index for functional assessment of arterial stiffness in mouse aortas, which may lead to controversy.

In conclusion, our findings suggest that C/EBPβ activation in VSMCs plays a critical role in the development of arterial stiffness induced by a high-fat diet. This process is linked to the regulation of VSMC phenotypic transition upon C/EBPβ activation. Additionally, C/EBPβ activation promotes the secretion of PDGF-CC, and we observed a positive correlation between PDGF-CC levels and arterial stiffness in humans. These results uncover a novel molecular mechanism behind arterial stiffness in the context of hyperlipidemia, offering potential therapeutic targets and biomarkers for managing conditions related to arterial stiffness.

## Materials and methods

### Animals

ApoE^−/−^ mice were obtained from Vital River Laboratory Animal Technology (Beijing, China). VSMCs C/EBPβ conditional knockout mice (C/EBPβ^fl/fl-^-Tagln^cre^ mice) and ApoE^−/−^ mice with VSMCs C/EBPβ conditional knockout mice (ApoE^−/−^/C/EBPβ^fl/fl-^-Tagln^cre^) were both constructed by Cyagen Biotechnology (Jiangsu, China). All mice used were eight weeks old, with the exception of the females indicated in the figure legend (Supplementary Fig. 3a, b), the remaining mice were male. All mice were housed in the West China Animal Experimental Center at the Chengdu Frontier Medical Center, and all housing conditions met the requirements for breeding genetically modified mice. The temperature in the animal facility was maintained at 22 °C ± 2 °C, with humidity kept between 40% and 60%. The light cycle followed the natural day-night rhythm. No more than five mice were housed per cage, and sterile bedding was used with independent ventilation systems. We conducted regular health checks on the mice and performed genetic testing for knockout mice after they reached adulthood, the genes and conditions used for identification are provided in Supplementary Fig. 5. Based on our experimental design, different types of feed were provided to the mice after reaching adulthood. All diets were obtained from Xietong Biological Engineering (Jiangsu, China). Mice were divided into different dietary groups, including a control group fed on a normal chow diet (control group, CAT No.1010001), an HFHC group fed on a high-fat, high-cholesterol diet (40% fat, 1.25% cholesterol, CAT No. XT108C, atherosclerotic diet), and an HF group fed on a high-fat diet (60% fat, CAT No. XTHF60). After a specific time of feeding mice were euthanized by cervical dislocation, and the aorta and blood samples were collected.

### Cells

Primary aortic VSMCs were isolated from mouse aorta using collagenase-elastase digestions. Dulbecco’s modified eagle’s medium (DMEM), penicillin-streptomycin, L-glutamine, non-essential amino acids and sodium pyruvate were obtained from Gibico (CarIsbad, CA, United States). All DMEM was supplemented with 10% fetal bovine serum (FBS, Gibico), and the cells were cultured in a humidified incubator at 37 ^◦^C with 5% CO_2_. MBD-Chol was obtained from Sigma (MO, USA). Following established protocols from previous investigations, we employed MBD-Chol at a concentration of 10 μg/mL with a 72-hour stimulation period to induce phenotypic transformation of contractile vascular smooth muscle cells (VSMCs) into macrophage-like cells^14^, this standardized treatment condition was consistently applied throughout our experimental procedures for all MBD-Chol cellular interventions.

### Aortic pulse wave velocity and Aortic contractile function

Aortic PWV (mm/ms) was measured using Doppler ultrasound (Vevo 3100, Fujifilm Visualsonics, Toronto, ON, Canada) following a previously established method^44^. Mice were anesthetized by inhaling 3% isoflurane mixed with air and maintained under light anesthesia (0.5-1% isoflurane/air) throughout the procedure. The mice were positioned in a supine posture on a heated platform (38 °C), with their paws in contact with electrode pads for continuous electrocardiogram (ECG) monitoring. Eye lubricant was applied to prevent corneal damage due to the loss of the blink reflex during anesthesia. To measure aPWV, a cross-sectional image of the abdominal aorta was first captured at the level of the renal vein, which served as the distal site for blood flow. The scan was then rotated by 90° to visualize the root of the ascending aorta as the proximal site. Flow waveforms were consecutively recorded at both the proximal and distal sites using Doppler mode. The arrival times, averaged over 10 cardiac cycles, were determined by measuring the distance between the foot of the flow waveform and the R-wave of the ECG at both locations using the Vevo3100 software. To calculate the distance between the two sites, the entire aorta, from the base of the aortic root to the iliac bifurcation, was carefully dissected in mice (Supplementary Fig. 6). The distance was then measured in vitro and used to compute the aPWV by dividing the total distance (d) by the difference in arrival times (transit time).

Aortic contractile function was quantified using the vasoactive diameter ratio (minimum diameter during vasoconstriction [Dmin] / maximum diameter during vasodilation [Dmax]). Triplicate measurements were obtained from three standardized anatomical landmarks along the aortic arch. All data analyses were conducted using the Vevo 3100 software (Fujifilm Visualsonics, Toronto, ON, Canada).

### Modified Russell–Movat’s Pentachrome stain and oil red O stain

After collecting aorta samples, paraffin sections were prepared and stained using the Movat Pentachrome Stain Kit (MPS-2 Kit, ScyTek Laboratories, Utah, USA) following the manufacturer’s protocol. For oil red O staining, aortas were harvested from the base of the ascending aorta to the iliac bifurcation. Following complete dissection, the aortas were fixed in 10% neutral-buffered formalin (NBF) for a minimum of 24 h. The samples were then stained with oil red O (ORO, Beyotime, Shanghai, China). After longitudinal dissection, the specimens were pinned onto wax plates for the identification and quantification of ORO+ plaques using light microscopy. For cell oil red O staining, cells were washed with PBS and fixed in 4% paraformaldehyde for 10–15 min. The cells were incubated with freshly filtered oil red O solution for 20 min at room temperature, then briefly rinsed with 60% isopropanol to remove excess stain. Nuclei were counterstained with hematoxylin, followed by rinsing with water and mounting with glycerol gelatin. The stained cells were examined under a bright-field microscope. Quantification of elastin fibers, proteoglycans, and plaque area was performed using ImageJ software (version 1.8.0).

### Immunofluorescence

Cells were seeded in 24-well plates containing poly-L-lysine-coated coverslips at a density of 2 × 10^5^ cells/ml. After the designated treatment, the coverslips were washed three times with PBS, fixed with 4% paraformaldehyde for 15 min at room temperature, and then permeabilized with 0.1% Triton X-100. After washing, the slides were incubated overnight at 4 °C with primary antibodies against C/EBPβ (Santa Cruz Biotechnology, Texas, USA; sc-7962), CD68 (Proteintech Group, Hubei, China; 28058-1-AP), or ACTA2 (Bioss, Beijing, China; ab124964). The following day, the slides were incubated with Cy3-conjugated goat anti-rabbit IgG or Alexa Fluor 488-labeled goat anti-mouse IgG (Beyotime) for 1 h at room temperature. DAPI solution was then applied to the slides for 5 min. The slides were sealed with an anti-fluorescence quenching agent (Beyotime) and examined using a Leica Stellaris confocal laser scanning microscope.

### Blood lipid levels testing

Whole blood was collected from mice and set aside at room temperature for one hour. Then the supernatant (serum) was obtained by centrifuging the whole blood at 3000 g for 10 min. The serum samples were used to measure the levels of four lipid parameters by an automatic biochemical analyzer (Mindray, BS-120, Guangzhou, China), including TG, TC, LDL-c, HDL-c.

### Small interfering RNA (si-RNA) transfection

C/EBPβ and scrambled siRNAs were obtained from Shenggong Corp (Shanghai, China). VSMCs were cultured in 12-well plates for 12 h and then transfected with a mixture containing 4 μl of Lipofectamine 2000 (Invitrogen, Carlsbad, CA, United States) and 3 μl of siRNA in 125 μl of Opti-MEM (Invitrogen). After another 12 h of incubation, the medium was changed to DEME containing 0.5 mg/ml cholesterol crystals or PBS, and the cells were further cultured for 24 h. The sequence numbers of the knocked-down genes can be found in Supplementary Table 4.

### Quantitative real-time polymerase chain reaction

Total RNA was extracted from treated VSMCs using RNAiso reagent (Takara, Ohtsu, Japan). The extracted RNA was then reverse transcribed into cDNA using the PrimeScript RT reagent Kit (Takara) following the manufacturer’s guidelines. Quantitative PCR was performed using the SYBR Premix Ex Taq II kit (Takara). The primer sequences used are provided in Supplementary Table S4.

### Western blot analysis (WB)

VSMCs were harvested and homogenized in lysis buffer, supplemented with protease and phosphatase inhibitors. The lysates were incubated on ice for 30 min, and protein concentration was determined using the BCA assay. The whole-cell lysates were separated by SDS-PAGE and transferred to PVDF membranes. Membranes were blocked with 5% non-fat milk in Tris-buffered saline with Tween-20 (TBST) at room temperature for 2 h. The membranes were then incubated overnight at 4 °C with primary antibodies against C/EBPβ (Abcam, MA, USA; ab32358), GAPDH (Abcam, ab9485), CD68 (Proteintech Group; 28058-1-AP), ACTA2 (Abcam, ab5694), LGALS3 (CST, MA, USA; #87985), PDGF-CC (Abclonal; A15174), TNFSF11 (CUSBIO; CSB-PA003937), and DAAM1 (Proteintech Group; 14876-1-AP). After washing three times with TBST, the membranes were incubated with an HRP-conjugated secondary antibody (Beyotime) at 37 °C for 2 h. Protein bands were detected using chemiluminescence (ChemiDoc MP, Bio-Rad).

### Chromatin Immunoprecipitation sequencing

The procedures of ChIP-seq can be found in our previous studies^25^. ChIP kits were purchased from Thermo Scientific (MA, USA). Both the control and Methyl-β-cyclodextrin cholesterol (MBD-Chol) groups included the following: input (positive control), IgG (negative control), and anti-C/EBPβ (target) groups. For positive control IP, 5 μg of H3K27ac antibody (Abcam) was used, and for the negative control IP, 5 μg of normal rabbit IgG was used. The target-specific IP used 5 μg of C/EBPβ antibody (Santa, sc-7962). The ChIP procedure was carried out as previously described. After the ChIP experiment, the ChIP-seq library was constructed. The results were visualized using the Integrative Genomics Viewer (IGV) and Model-based Analysis of ChIP-seq (MACS). ClusterProfiler software was used for enrichment analysis of Gene Ontology (GO), Kyoto Encyclopedia of Genes and Genomes (KEGG), and Reactome databases. Motif sequences were identified using Homer software and compared with the JASPAR database. The relevant Flow chart for ChIP-seq is provided in Supplementary Fig. 7.

### Ingenuity pathway analysis

We used Ingenuity Pathway Analysis (IPA, QIAGEN Digital Insights, CA, USA) to analyze the data obtained from ChIP-seq. The database included Ingenuity Expert Information and Ingenuity Supported Third Party Information. The detailed filtering criteria are shown in the Supplementary Fig. 8.

### Cytoskeleton staining

The cytoskeleton was stained with Coomassie blue and Phalloidin to assess the impact of C/EBPβ on cytoskeleton changes during the phenotypic transition of VSMCs. The kits used were obtained from Bestbio Biological Company (Jiangsu, China. Cat No. BB-4440, and Cat No. BB-44402). The experiments were performed according to the manufacturer’s instructions. The quantification of Phalloidin area was performed using ImageJ software (1.8.0).

### Flow cytometry

Isolate aortic cells from mice using enzymatic digestion (collagenase IV + DNase I). Filter through a 70-µm cell strainer and resuspend in PBS + 2% FBS. Add Fixable Viability Stain 620 (BD Pharmingen, 564996) to cells for viability Staining. Incubate for 15 min at 4 °C in the dark. Add Fc Block (Thermo Fisher Scientific) for 10 min at 4 °C. Stain with anti-CD68-Pacific Blue (BioLegend, 137028) for 30 min at 4 °C.Wash twice with PBS + 2% FBS. Using Foxp3 Fixation Buffer (from the Foxp3 kit) for 30 min at 4 °C. Permeabilize with Foxp3 Permeabilization Buffer for 1 h at 4 °C. 5. Stain with anti-ACTA2-APC (R&D Systems, IC1420A) in Permeabilization Buffer for 1 h at 4 °C. Wash twice with Permeabilization Buffer. Add anti-C/EBPβ-Alexa Fluor® 488 (Abcam, ab237414) in Permeabilization Buffer. Incubate overnight at 4 °C (enhances nuclear target penetration). Wash twice and resuspend in PBS + 2% FBS. Exclude dead cells during gating. Use sequential gating: Cells→ Live cells → CD68-positive or ACTA2-positive or CD68 and C/EBPβ double-positive cells. Sample detection and analysis were performed using the NovoCyte system (Agilent).

### Enzyme-linked immunosorbent assay (ELISA)

Human ELISA Kit was obtained from R&D Systems (MN, USA. RANKL, CAT No.DY626; PDGF-CC, CAT No.DY1687-05). PDGF-CC Mouse ELISA Kit was obtained from Sino-American biotechnology (Hubei, China. CAT No. CSB-E13404m). TNFSL11 Mouse ELISA Kit was obtained from Elabscience Biotechnology (Hubei, China. CAT No. E-EL-M0644c). ELISA was performed according to the manufacturer’s instructions. The tests were performed using human serum samples and mouse VSMCs supernatant.

### Analysis of RNA-sequencing data

External RNA sequencing data used in this study were obtained from a previous study^26^ and downloaded from the Gene Expression Omnibus (GEO) database at http://www.ncbi.nlm.nih.gov/geo. The accession number for the dataset was GSE163244 (https://ncbi.nlm.nih.gov/geo/query/acc.cgi?acc=GSE163244). The sequencing platform used for this dataset was Illumina HiSeq 2000 (Homo sapiens). The gene expression matrix was annotated using the reference genome GRCh38.p13 (www.gencodegenes.org/human/). Volcano plots were generated using GraphPad Prism 9 (GraphPad Software, CA, USA).

### Population study participants

We carried out this cross-sectional study from January to December 2021. Adults aged 18 and older were recruited from two locations in Sichuan Province, China, as part of a nationwide survey on cardiovascular diseases and associated risk factors among Chinese residents. Participants using antihypertensive, lipid-lowering, or hypoglycemic medications were excluded. Additionally, individuals with a history of stroke, heart failure, coronary artery disease or peripheral arterial disease (ankle-brachial index [ABI] < 0.9) were also excluded. The final dataset included individuals with complete medical records, including demographic data, medical history, personal habits, blood biochemistry, and baPWV. To reduce selection bias, we adjusted for variables such as age, sex, body mass index (BMI), smoking and alcohol consumption using 1:2 propensity score matching, based on baPWV ≥1400 cm/s and baPWV < 1400 cm/s. Ultimately, 360 participants were included in the analysis. The study was conducted in accordance with the ethical principles outlined in the Declaration of Helsinki^45^.

### Physical examination and medical history collection

Basic individual information, medical history, and chronic disease medication data, including hypertension and diabetes mellitus, were collected by trained investigators. Physical examinations, including height, weight, and blood pressure (BP), were conducted in a quiet room maintained at approximately 25 °C. Office BP measurements were taken using validated electronic sphygmomanometers (OMRON HEM-7200). Participants were asked to rest for 5 min before the measurement. Systolic and diastolic BP were recorded three times on the right arm while the participant was seated. The average of the three readings was used for analysis. Blood samples were collected in the morning after 8 h of overnight fasting. Fasting blood glucose (FBG), TG, TC, HDL-c, LDL-c were measured using an automatic biochemical analyzer. Plasma PDGF-CC concentration was measured by Servicebio Technology Co., Ltd. (Wuhan, China). BaPWV was measured by trained physicians using an automated device (BP-203RPEII, Nihon Colin, Japan), as described previously^46^. Prior to the measurement, participants were asked not to consume alcohol for 24 h and to refrain from smoking, eating, or drinking irritant beverages including tea or coffee for 2 h. The operator applied an inflated cuff to the upper and lower limbs, attached electrodes of the ECG device to both wrists, and placed the heart sound sensor at the left sternal border. The device automatically recorded and calculated baPWV values for both the left and right side. The mean values of the baPWV from both sides were used for analysis. In this study, high baPWV was defined as baPWV ≥ 1,400 cm/s, as baPWV ≥ 1400 cm/s is an independent variable for risk stratification by Framingham score and serves as an evaluation parameter for early target organ damage in hypertension^47,48^.

### Ethics statements

All animal experiments were approved by the Animal Ethics Committee of West China Hospital and were conducted in accordance with relevant regulatory guidelines. The study involving human participants was approved by the ethics committee of West China Hospital, Sichuan University, and registered with the Chinese Clinical Trial Registry (registration number: ChiCTR2100054493).

### Statistical analysis

For experimental studies, statistical analysis was conducted using SPSS 26.0 software (IBM, Inc., Armonk, NY, USA). Results were presented as mean ± standard error of the mean (SEM). Two-group comparisons were performed using an unpaired t test, while multiple comparisons were carried out using one-way analysis of variance (ANOVA) followed by Tukey’s studentized range (HSD) test. A *p*-value of <0.05 was considered statistically significant, and a *p*-value of <0.01 was considered highly significant.

For population studies, SPSS 26.0 (IBM, Inc., Armonk, NY, USA) was used for data analysis. Continuous data with a normal distribution were expressed as means ± standard deviations (SD), while those with a non-normal distribution were represented as medians [interquartile range (IQR)]. Categorical variables were expressed as frequencies (%). Continuous data were compared using either the independent-samples *t* test or the Mann–Whitney *U* test. The chi-square test was applied to assess differences in categorical variables between groups. Bivariate Spearman’s correlation and partial correlation analysis were performed to explore the relationship between plasma PDGF-CC concentration and baPWV. Receiver operating characteristic (ROC) curves were used to evaluate the ability of plasma PDGF-CC levels to distinguish arterial stiffness (baPWV ≥ 1400 cm/s). The area under the ROC curve, cut-off values, Youden index (YI), sensitivity, specificity, positive predictive values (PPV), and negative predictive values (NPV) were also calculated. A *p*-value of <0.05 was considered statistically significant.

## Supplementary information

Supplementary Materials

Supplementary Table S5

## Data Availability

The raw ChIP-seq data can be downloaded from the Genome Sequence Archive (GSA) website (https://ngdc.cncb.ac.cn/gsa/), BioProject:PRJCA035824, BioSample:SAMC4669859:C/EBP-chip.
