# Supplementary material for: C/EBPβ activation in vascular smooth muscle cells promotes hyperlipidemia-induced phenotypic transition and arterial stiffness
Source: Signal Transduct Target Ther. 2025 Apr 2;10:105. doi: 10.1038/s41392-025-02196-w (PMC11962150; doi:10.1038/s41392-025-02196-w)

Supplementary Materials for

C/EBPβ activation in vascular smooth muscle cells promotes hyperlipidemia-induced phenotypic transition and arterial stiffness

Jun Ma^#^, Xiangyu Yang^#^, Yanan Li^#^, Xin Zhang^#^, Kai Liu, Yong Peng, Si Wang, Rufeng Shi, Xingwei Huo, Xueting Liu, Xinran Li, RunyuYe, Zhipeng Zhang, Changqiang Yang, Lu Liu, Dan Gao, Shanshan Jia, Mengzuo Xu, Lirong Sun, Xianghao Zuo, Qingtao Meng*, Xiaoping Chen*

Correspondence to: xiaopingchen23@163.com

**This PDF file includes:**

Figures. S1 to S9

Tables. S1 to S4

Uncropped Images of Western Blots

Other Supplementary Materials for this manuscript include the following:

Supplementary Tables.S5 Functional annotation and classification of genes that C/EBPβ can bind to in the ChIP-seq results.


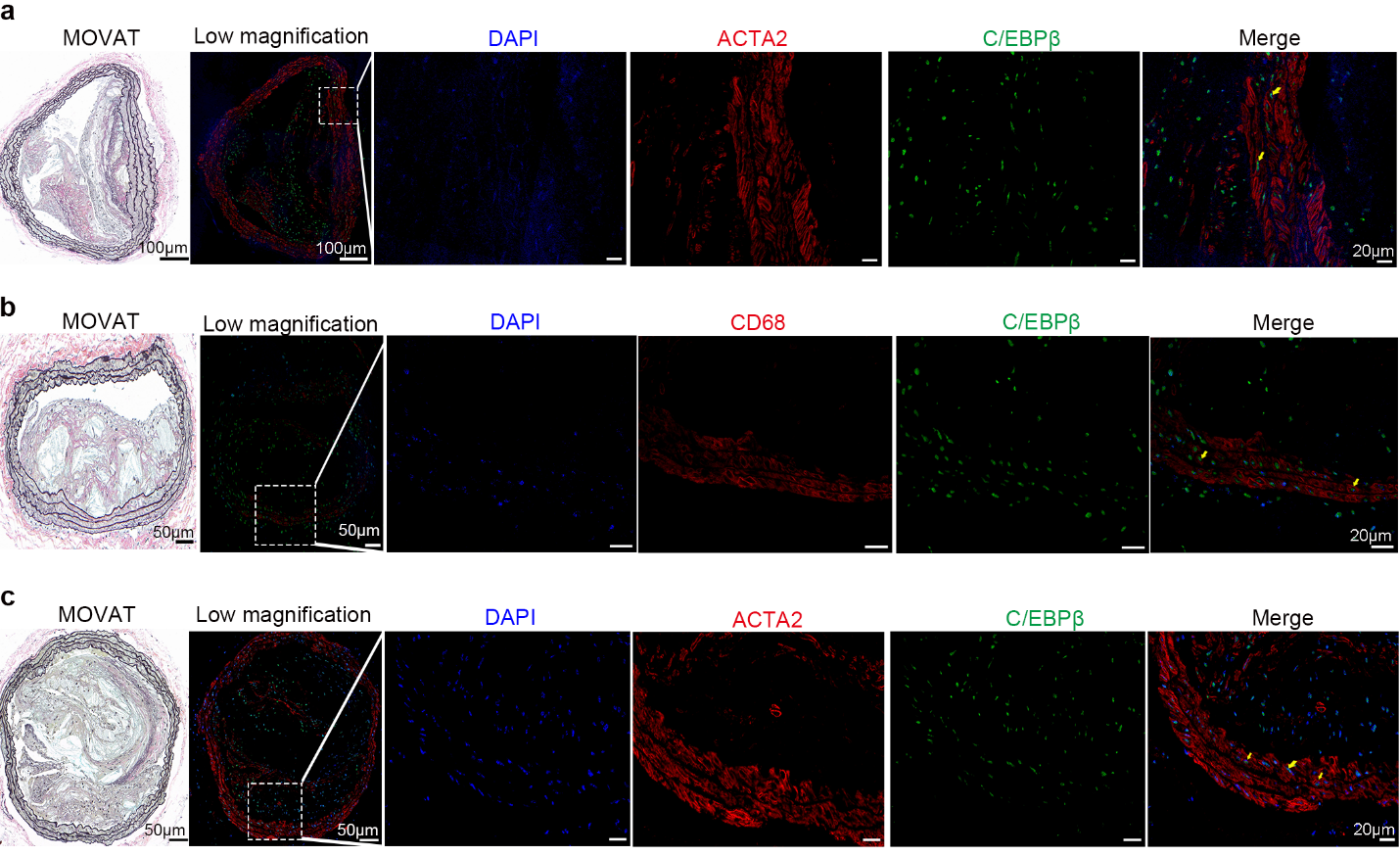


**Figure. S1. C/EBPβ is expressed in the vascular walls of atherosclerotic arteries induced by a high-fat diet.**

**a** Fluorescent colocalization of ACTA2 and C/EBPβ in the aortic arch. **b** Fluorescent colocalization of CD68 and C/EBPβ in the abdominal aorta. **c** Fluorescent colocalization of ACTA2 and C/EBPβ in the abdominal aorta. Modified Russell-Movat Pentachrome stain was used to show murine aortic arch tissue composition, black for elastin fibers, yellow for collagen, blue/cyan for proteoglycan, red for collagen fibers, and black/purple for nuclei. Immunofluorescence staining was used to assess protein expression and localization in the murine aortas, blue (DAPI) for nuclei, green for C/EPBβ, and red for CD68 or ACTA2. The scale bars correspond to 100 μm for low-magnification images and 20 μm for high-magnification views.


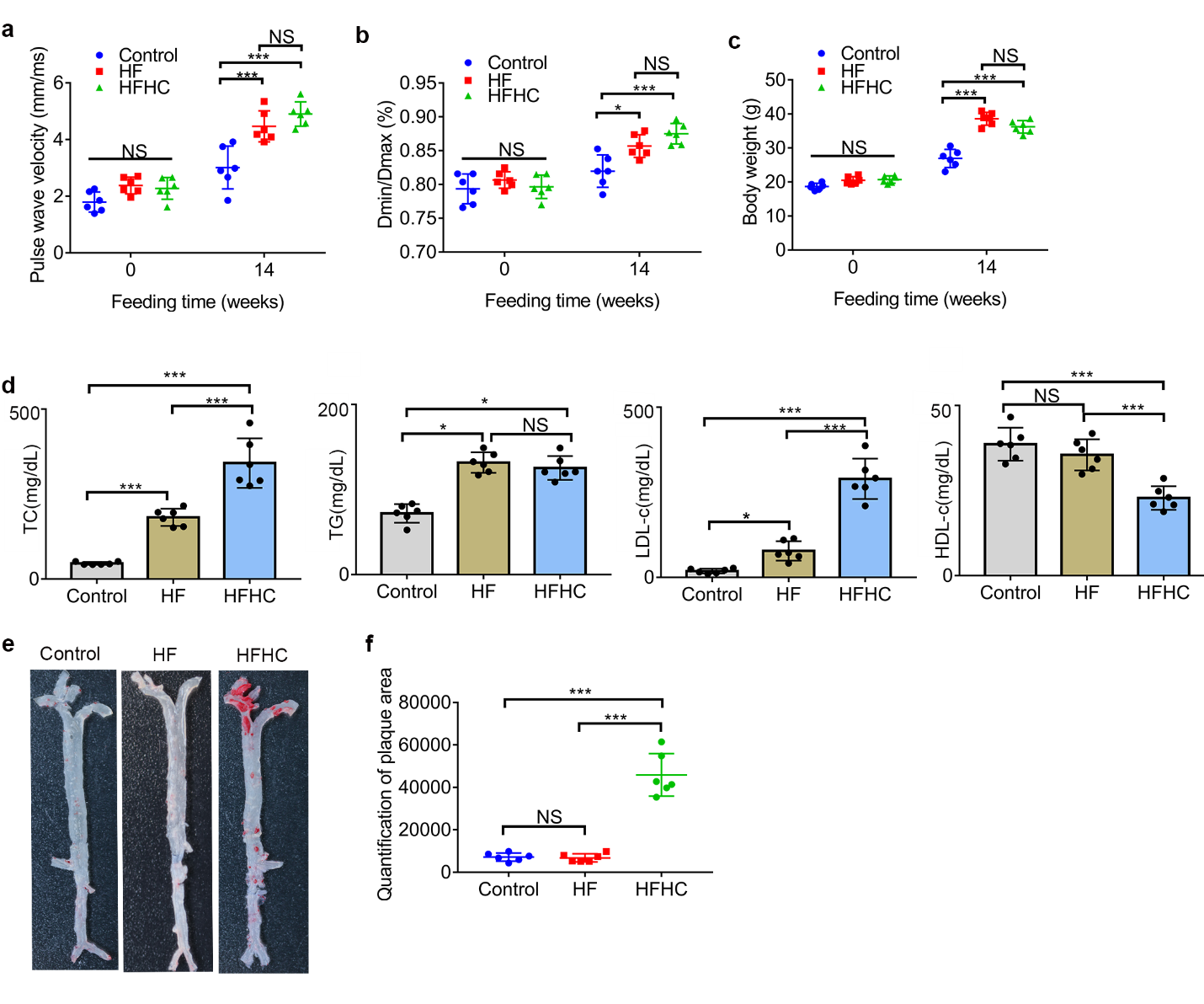


Figure. S2. Changes in lipids levels, arterial stiffness and plaque formation in the HF group and the HFHC group.

**a** Statistical results of changes in aPWV, N=6. **b** Statistical results of aortic contractile function, N=6. **c** Changes in body weight, N=6. **d** Changes in blood lipids levels, N=6. **e** Plaque formation in different groups (representative images). **f,** Statistical results of plaque formation in different groups, N=6. Control=ApoE^-/-^ mice fed with chow food, HF=ApoE^-/-^ mice fed with a 60% high-fat diet, HFHC=ApoE^-/-^ mice fed with a 40% fat and 1.25% cholesterol diet. TC=total cholesterol, TG=triglycerides, LDL-c=low-density lipoprotein cholesterol, HDL-c=high-density lipoprotein cholesterol, NS= nonsense, * p <0.05 and *** p <0.001.


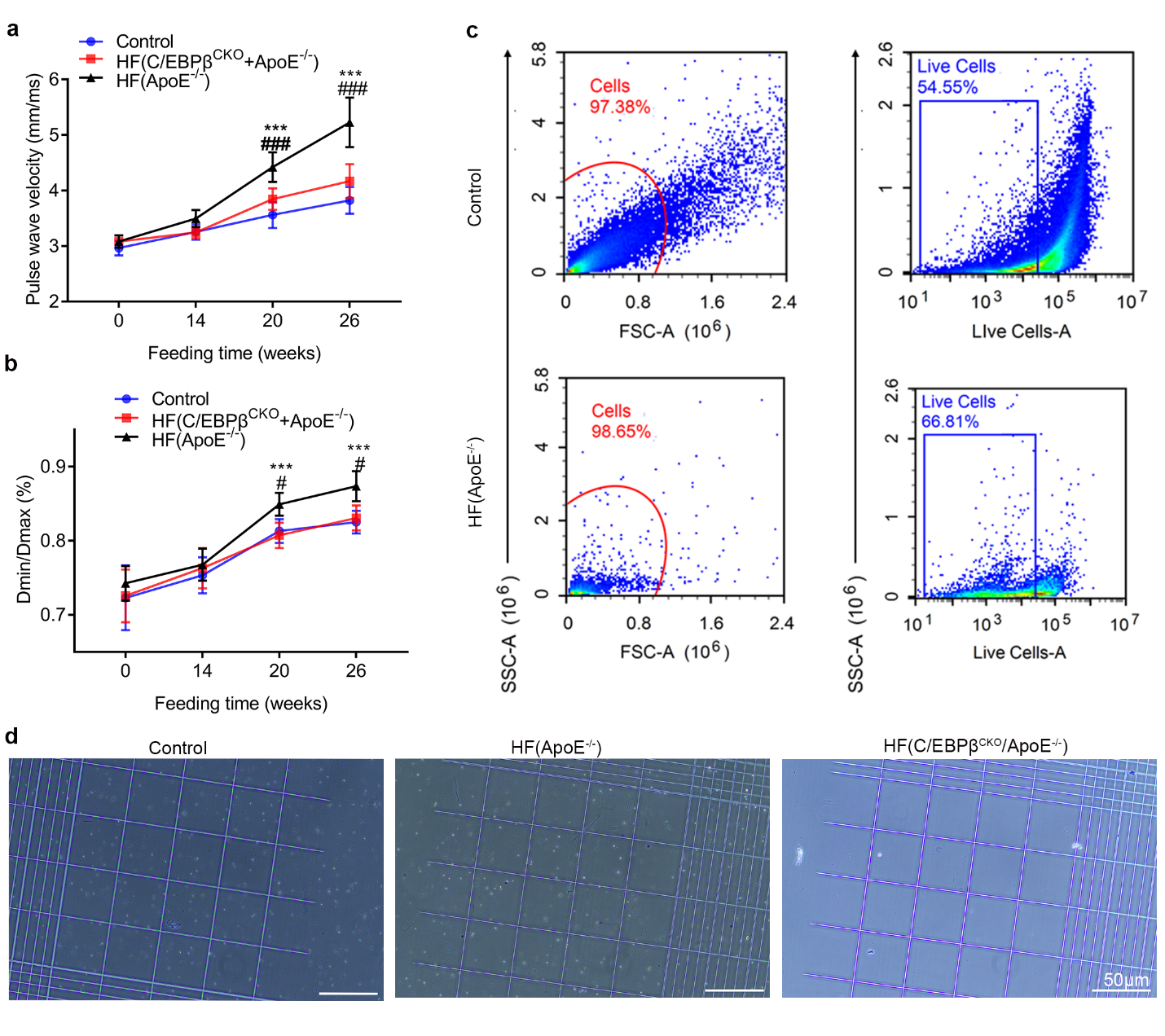


Figure. S3. Supplementary results relevant to the study.

**a** Changes in aPWV across different treatment groups of female mice, N=6. **b** Aortic contractile function in different treatment groups of female mice, N=6. **c** Supplementary flow cytometry results of the Control group and HF (ApoE^-/-^) group. **d** The number of viable cells obtained from digested aortas of mice in each group and the genes & reads detection results from single-cell sequencing. Control=ApoE^-/-^ mice fed with chow food, HF (ApoE^-/-^) =ApoE^-/-^ mice fed with a 60% high-fat diet, HF (C/EBPβ^CKO^/ApoE^-/-^) =ApoE^-/-^/C/EBPβ^fl/fl-^-Tagln^cre^ mice fed with a 60% high-fat diet. * p <0.05 and *** p <0.001 compared with 0-week, # p <0.05 and ### p <0.001 compared with the control group.


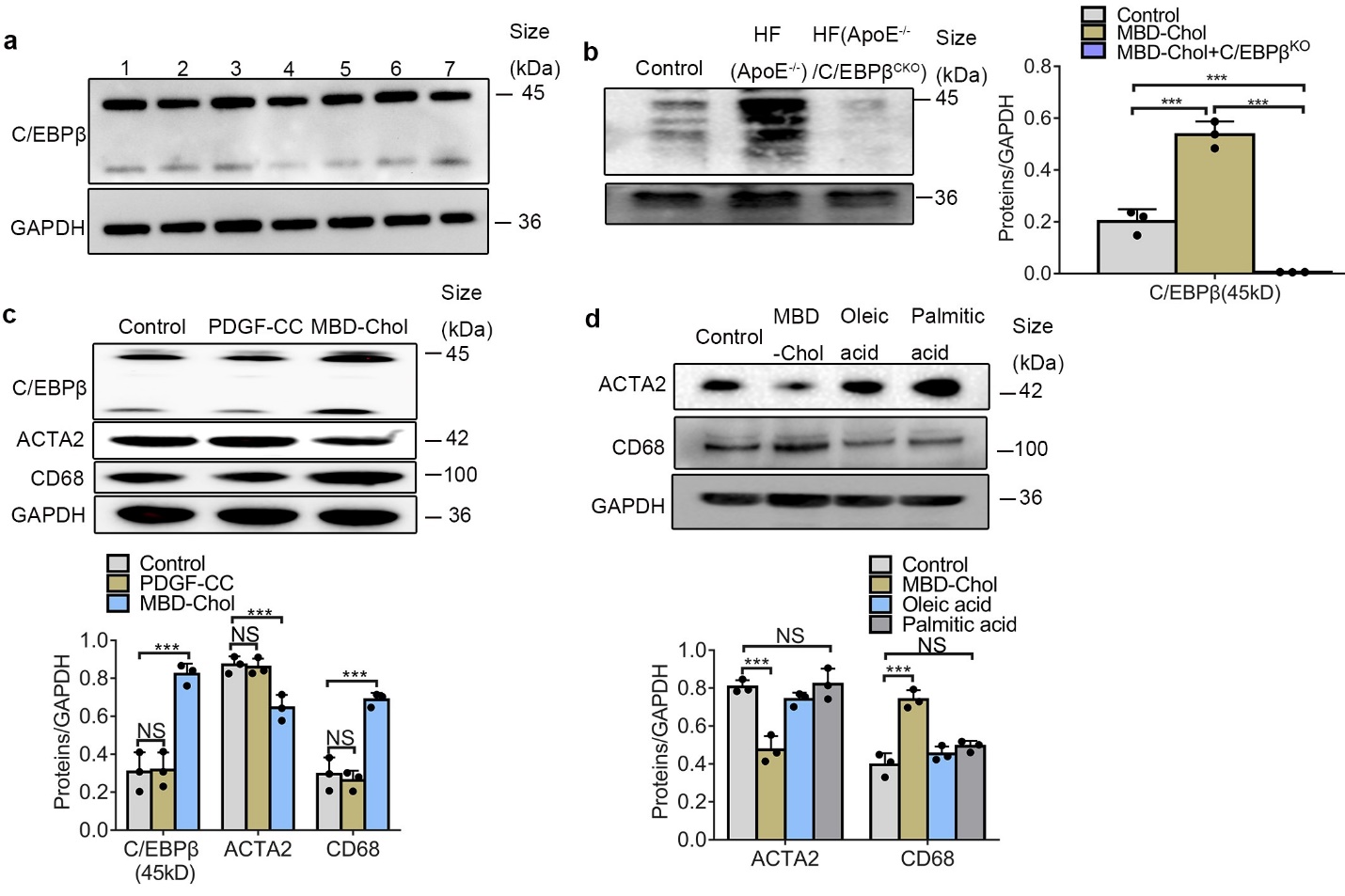


Figure. S4. Supplementary results relevant to the study.

**a** Protein content in bone marrow-derived macrophages (BMDMs) detected by WB; Sample 1-6 were obtained from BMDMs from 6 C/EBPβ^CKO^/APOE^-/-^ mice, and Sample 7 was obtained from BMDMs from WT mice. **b** Extracted mouse aortic protein, with representative WB images and statistical graphs, N=3. **c**  Protein content in VSMCs after simulated by PDGF-CC or MDB-Chol (top, representative images); Quantification results of protein content in VSMCs (bottom，N=3). **d** Protein content in VSMCs following intervention of MBD-Chol (10 μg/ml), oleic acid (100 μM), and palmitic acid (100 μM) for 72h (top, representative images); Quantification results of protein content in VSMCs (bottom, N=3). NS= nonsense, * p <0.05 and *** p <0.001.

Table S1. Normality test for various variables in the population study.

| Variables | | K-S Test | | | S-W Test | | |
| --- | --- | --- | --- | --- | --- | --- | --- |
|  |  | D | df | *P* value | W | df | *P* value |
| Age | G1 | 0.093 | 120 | 0.013 | 0.979 | 120 | 0.062 |
|  | G2 | 0.119 | 240 | ＜0.001 | 0.973 | 240 | ＜0.001 |
| BMI | G1 | 0.088 | 120 | 0.023 | 0.957 | 120 | 0.001 |
|  | G2 | 0.280 | 240 | ＜0.001 | 0.239 | 240 | ＜0.001 |
| SBP | G1 | 0.094 | 120 | 0.011 | 0.974 | 120 | 0.019 |
|  | G2 | 0.072 | 240 | 0.004 | 0.983 | 240 | 0.005 |
| DBP | G1 | 0.069 | 120 | 0.200 | 0.973 | 120 | 0.017 |
|  | G2 | 0.041 | 240 | 0.0200 | 0.994 | 240 | 0.515 |
| FBG | G1 | 0.201 | 120 | ＜0.001 | 0.558 | 120 | ＜0.001 |
|  | G2 | 0.097 | 240 | ＜0.001 | 0.910 | 240 | ＜0.001 |
| TG | G1 | 0.170 | 120 | ＜0.001 | 0.817 | 120 | ＜0.001 |
|  | G2 | 0.208 | 240 | ＜0.001 | 0.715 | 240 | ＜0.001 |
| TC | G1 | 0.081 | 120 | 0.049 | 0.987 | 120 | 0.307 |
|  | G2 | 0.062 | 240 | 0.025 | 0.990 | 240 | 0.093 |
| HDL-C | G1 | 0.065 | 120 | 0.200 | 0.986 | 120 | 0.232 |
|  | G2 | 0.072 | 240 | 0.005 | 0.964 | 240 | ＜0.001 |
| LDL-C | G1 | 0.070 | 120 | 0.200 | 0.980 | 120 | 0.071 |
|  | G2 | 0.034 | 240 | 0.200 | 0.993 | 240 | 0.361 |
| CR | G1 | 0.227 | 120 | ＜0.001 | 0.466 | 120 | ＜0.001 |
|  | G2 | 0.069 | 240 | 0.008 | 0.984 | 240 | 0.010 |
| UA | G1 | 0.085 | 120 | 0.032 | 0.967 | 120 | 0.005 |
|  | G2 | 0.052 | 240 | 0.200 | 0.977 | 240 | 0.001 |
| PDGF-CC | G1 | 0.090 | 120 | 0.018 | 0.974 | 120 | 0.019 |
|  | G2 | 0.061 | 240 | 0.029 | 0.977 | 240 | 0.001 |
| BaPWV | G1 | 0.197 | 120 | ＜0.001 | 0.732 | 120 | ＜0.001 |
|  | G2 | 0.059 | 240 | 0.043 | 0.980 | 240 | 0.002 |

BMI, body mass index; SBP, systolic blood pressure; DBP, diastolic blood pressure; FBG, fasting blood glucose; TG, triglyceride; TC, total cholesterol; HDL-C, high density lipoprotein cholesterol; LDL-C, low density lipoprotein cholesterol; CR, Creatinine; UA, uric acid; PDGF-CC, Platelet derived growth factor CC; BaPWV, brachial-ankle pulse wave velocity.

Table S2. Spearman and partial correlation between PDGF-CC and BaPWV.

| **Model 1** | | | **Model 2** | | | **Model 3** | | |
| --- | --- | --- | --- | --- | --- | --- | --- | --- |
| correlation coefficient | *P* | correlation coefficient | | *P* | correlation coefficient | | *P* |  |
| 0.194 | ＜0.001 | 0.161 | | 0.002 | 0.148 | | 0.006 |  |

Model 1 unadjusted.

Model 2 age, gender, BMI, SBP, DBP, smoking, and drinking were controlled in partial correlation analysis.

Model 3 age, gender, BMI, SBP, DBP, smoking, drinking, FBG, TG, TC, LDL-C, HDL-C, CR, and UA were controlled in partial correlation analysis.

Table S3. The ROC curves analysis of PDGF-CC discriminating BaPWV≥1400cm/s.

| AUC 95%CI | Cut-off value | YI | PPV | NPV |
| --- | --- | --- | --- | --- |
| 0.608(0.546-0.670) | 826.02 | 0.2 | 0.446 | 0.740 |

AUC, area under the curve; ROC, receiver operating characteristic; CI, confidence interval; YI, Youden index; PPV, positive predictive values; NPV, negative predictive values.

**Table S4**

**Primer for qPCR**

| Gene | Forward primer (5’ -> 3’) | Reverse primer (5’ -> 3’) |
| --- | --- | --- |
| C/EBPβ | GCCAACTTCTACTACGAGCC | CGGAGAGGAAGTCGTGGTG |
| LGALS3 | AGGAGAGGGAATGATGTTGCC | GGTTTGCCACTCTCAAAGGG |
| CD68 | CAAGGTCCAGGGAGGTTGTG | CCAAAGGTAAGCTGTCCATAAGGA |
| ACTA2 | GCTTCGCTGGTGATGATGCTC | AGTTGGTGATGATGCCGTGTTC |
| CNN1 | TCTGCACATTTTAACCGAGGTC | GCCAGCTTGTTCTTTACTTCAGC |
| MYH11 | CATGGACCCGCTAAATGACA | CAATGCGGTCCACATCCTTC |

| **Sequence of siRNA** | | |
| --- | --- | --- |
| Gene | Sense (5’ -> 3’) | Anti-sense (5’ -> 3’) |
| C/EBPβ | GCGCAAGAGCCGAGAUAAATT | UUUAUCUCGGCUCUUGCGCTT- |
| Scrambled RNA | UCUAUAUCCACUUAUAGUCTT | GACUAUAAGUGGAUAUAGATT |


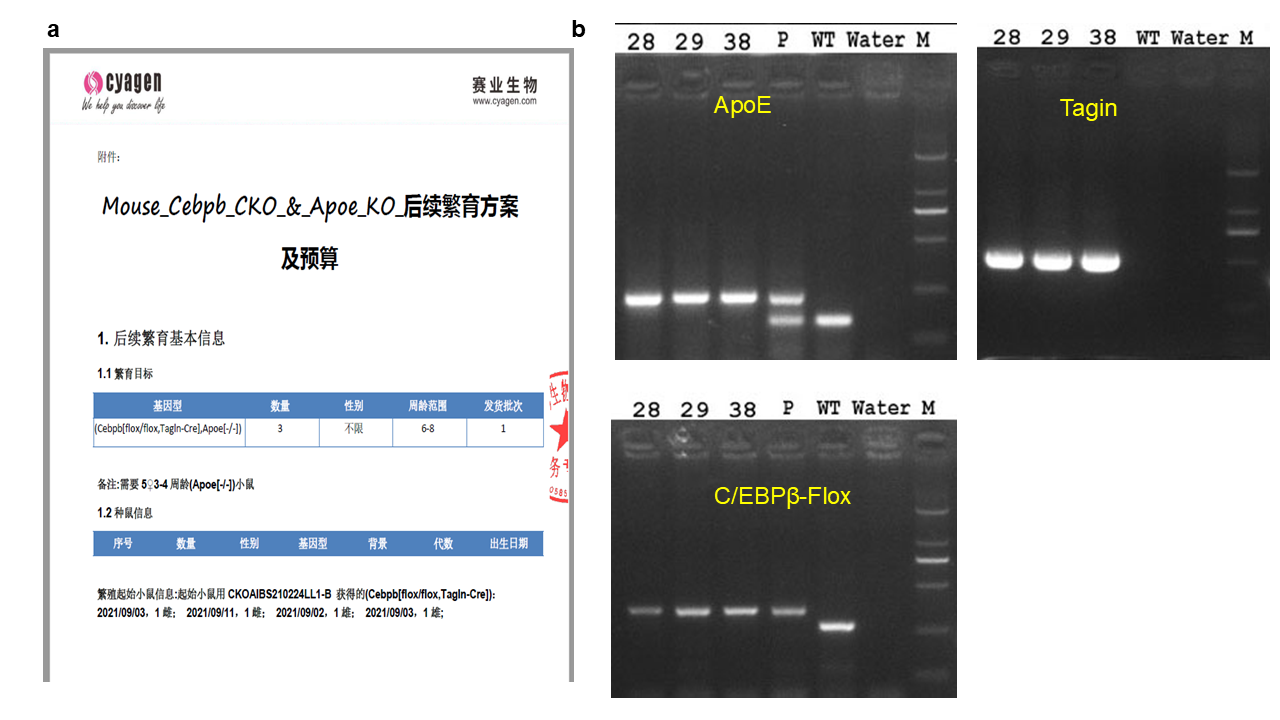


**Figure. S5. The construction of VSMCs C/EBPβ conditional and AopE^-/-^ double knockout mice.**

**a** Contract for constructing double knockout mice. **b** Identify the mouse genotype through tail DNA sampling before using the mice. The identification conditions are as follows:

Primers1: (Annealing Temperature 60.0 ºC)

C/EBPβ-Flox F1: 5’-GCCCTCTCGCGCTCCCGTGGCCG-3’

C/EBPβ-Flox R1: 5’-GTCGGGCTCGTAGTAGAAGTTG-3’

Homozygotes: one band with 352 bp

Heterozygotes: two bands with 352 bp and 284 bp

Wildtype allele: one band with 284 bp

Primers2: (Annealing Temperature 60.0 ºC)

Tagln-M-F: 5’-CAGACACCGAAGCTACTCTCCTTCC-3’

Tagln-M-R: 5’-CGCATAACCAGTGAAACAGCATTGC-3’

Cre amplicon: ~513 bp

Primers3: (Annealing Temperature 60.0 ºC)

APOE-M-F: 5’-GCCTAGCCGAGGGAGAGCCG-3’

APOE-M-R: 5’-GCCGCCCCGACTGCATCT-3’

APOE-W-R: 5’-TGTGACTTGGGAGCTCTGCAGC-3’

Homozygotes: one band with ~245 bp

Heterozygotes: two bands with ~245 bp and ~155 bp

Wildtype allele: one band with ~155 bp


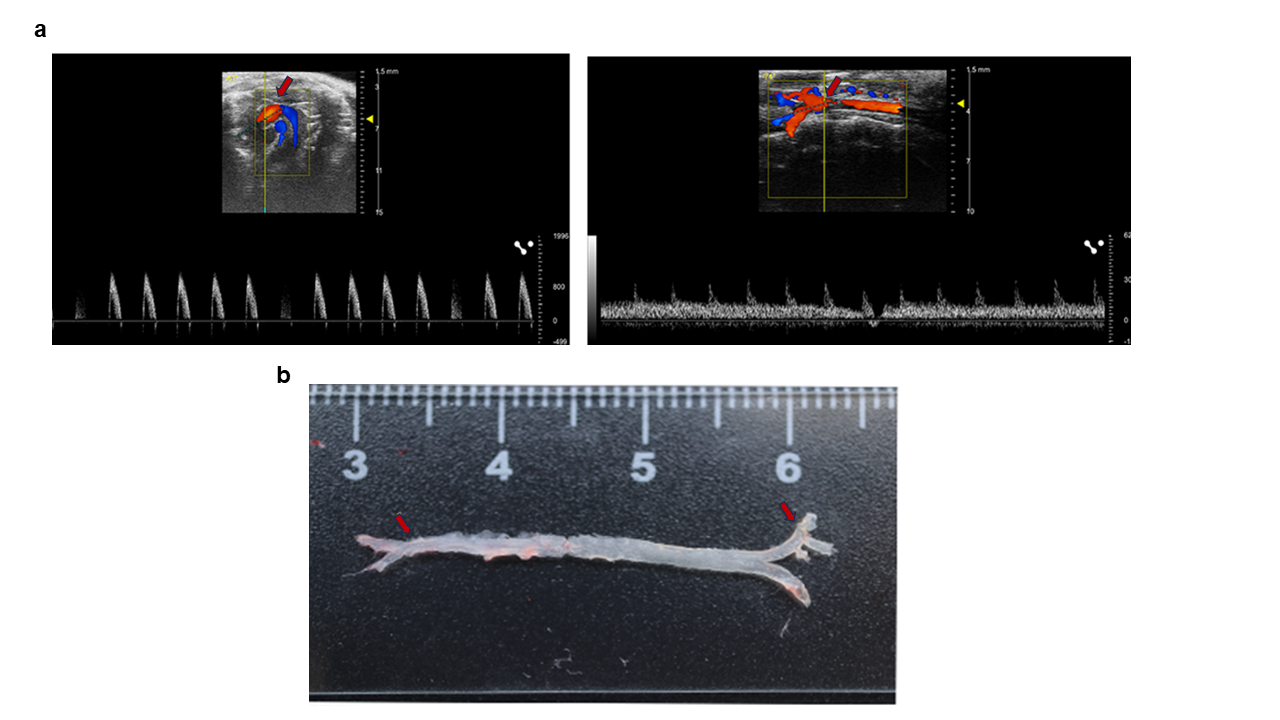

**Figure.S6. The anatomical sites for measuring aPWV**

**a,** Ultrasound imaging was used to locate the first branch of the ascending aorta and the bifurcation of the abdominal aorta in the mice (indicated by red arrows). **b,** In vitro measurement of the aortic length (the distance from the first branch of the ascending aorta to the bifurcation of the abdominal aorta).


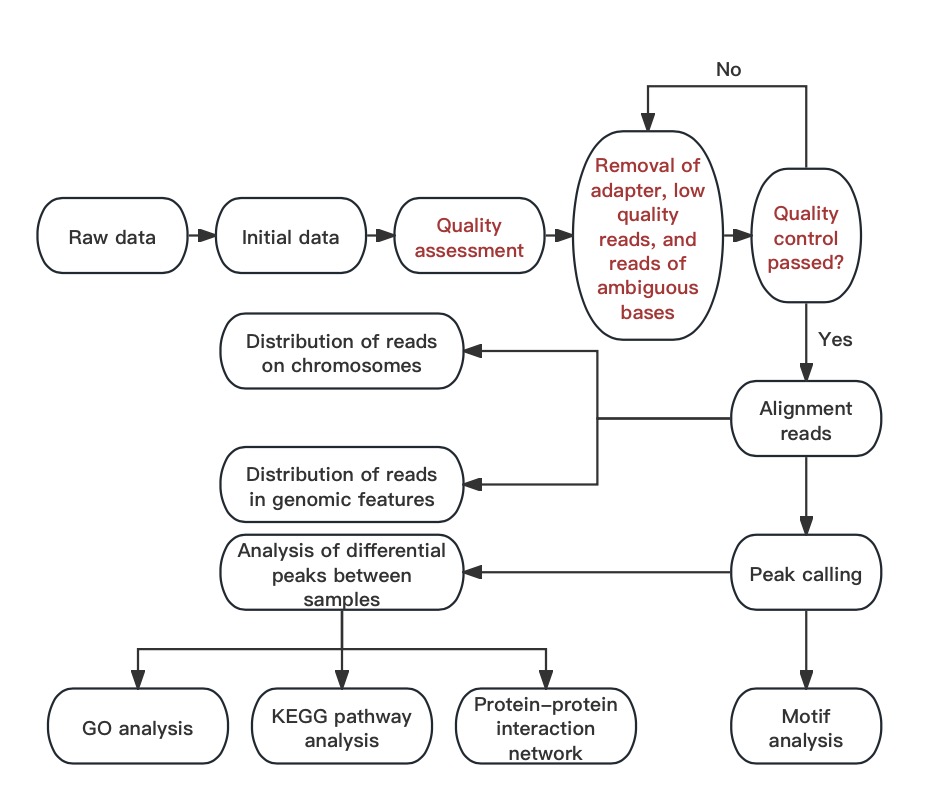


**Figure.S7. The workflow of ChIP-seq**


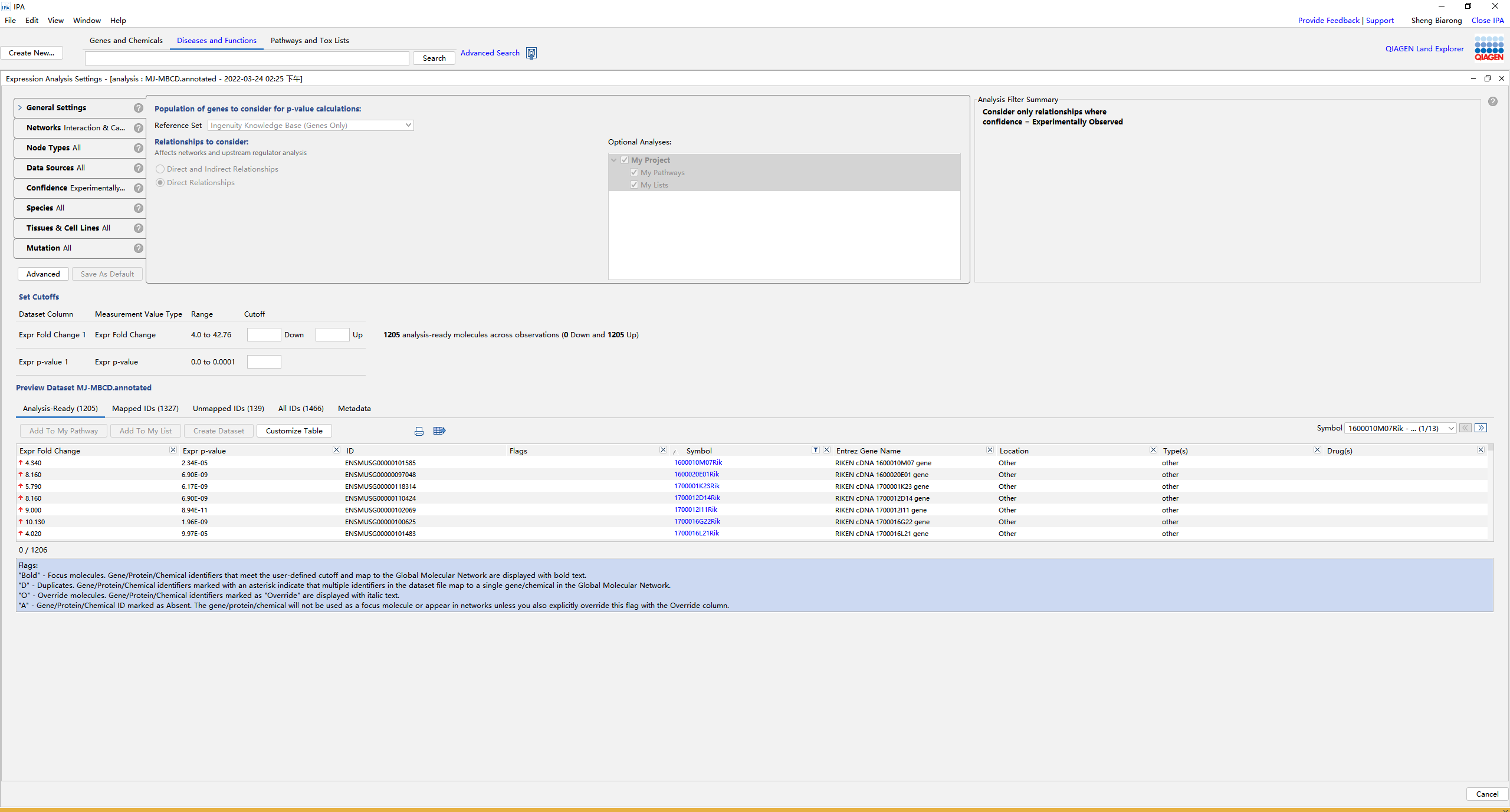


**Figure.S8.** **IPA analysis conditions for ChIP-seq**


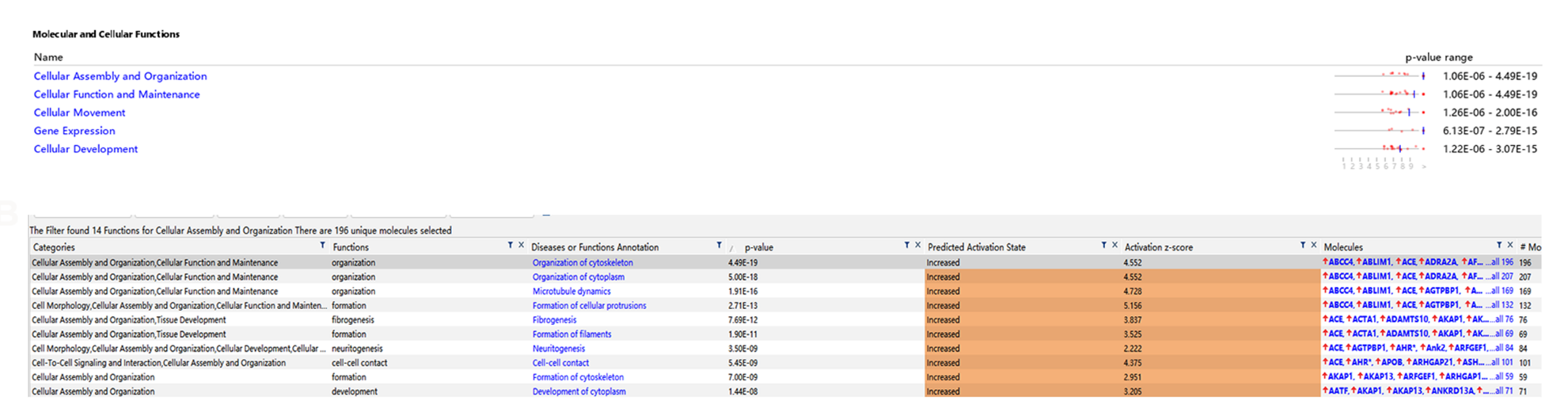


**Figure.S9.** **Original images of IPA. The upper image shows the analysis results of Molecular and Cellular Functions, the lower image shows the analysis results of Cell Assembly and Organization.**

**Uncropped Images of Western Blots**


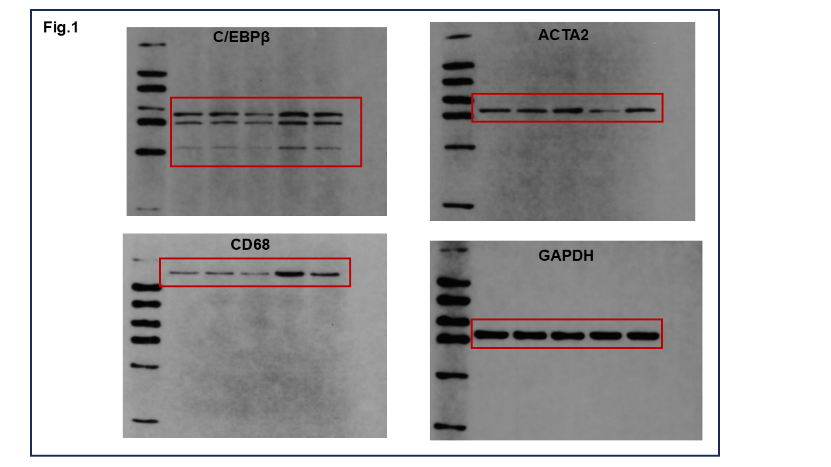

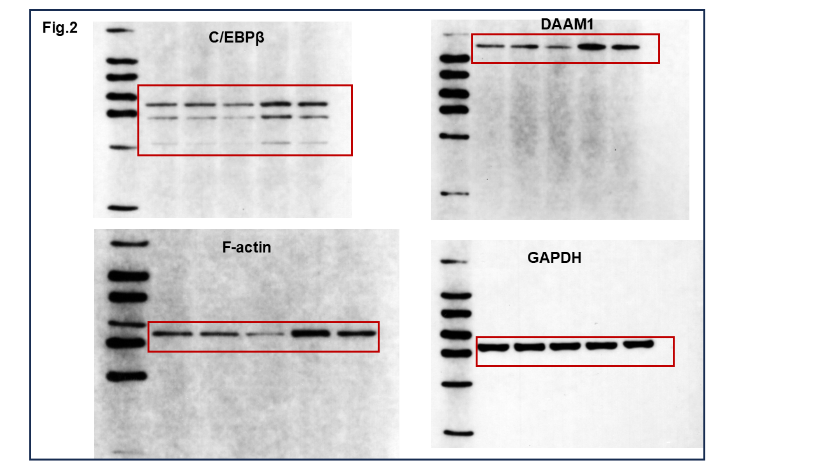

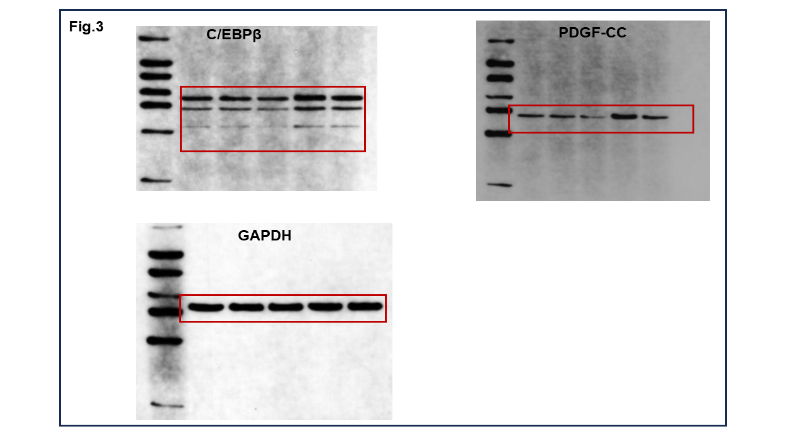

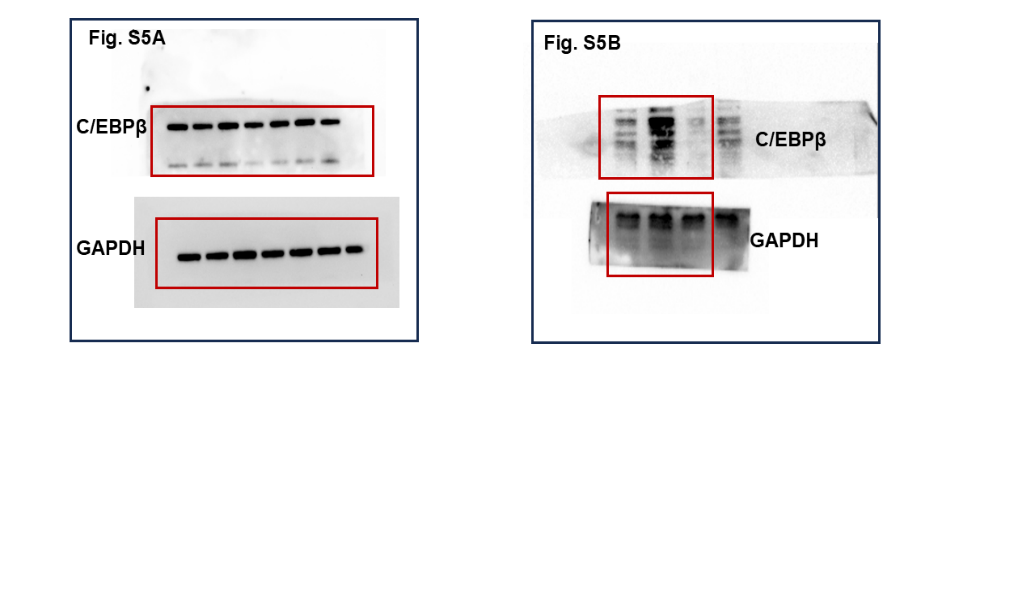

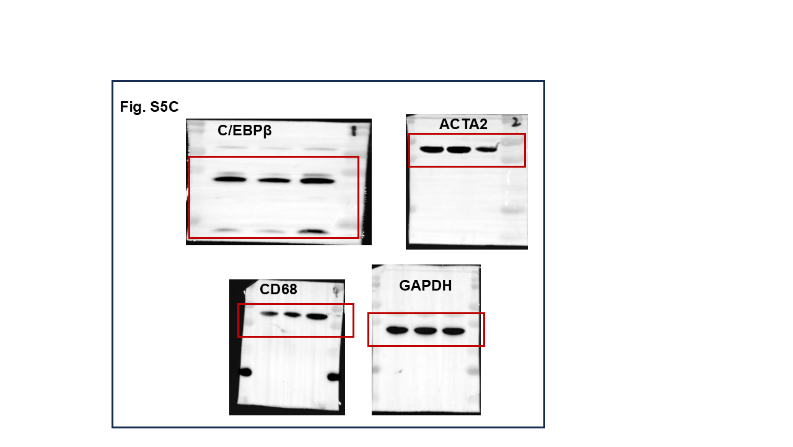


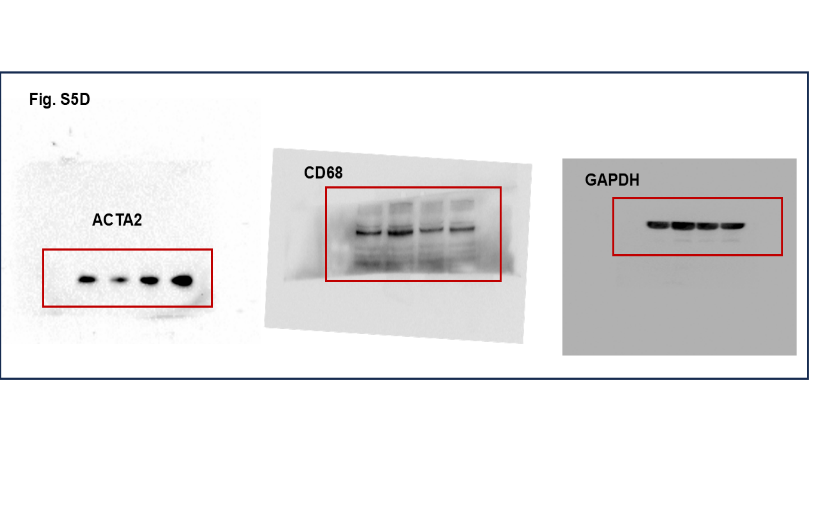

Supplement: Supplementary file 1 — Supplementary Materials [file 41392_2025_2196_MOESM1_ESM.docx]
